# Effects of short inter-pregnancy/birth interval on adverse perinatal outcomes in Asia-Pacific region: A systematic review and meta-analysis

**DOI:** 10.1371/journal.pone.0307942

**Published:** 2024-07-31

**Authors:** Tahir Ahmed Hassen, Melissa L. Harris, Desalegn Markos Shifti, Tesfalidet Beyene, Md Nuruzzaman Khan, Tesfaye Regassa Feyissa, Catherine Chojenta

**Affiliations:** 1 School of Nursing and Midwifery, College of Health and Medical Sciences, Haramaya University, Harar, Ethiopia; 2 Centre for Women’s Health Research, School of Medicine and Public Health, College of Health, Medicine and Wellbeing, University of Newcastle, Callaghan, NSW, Australia; 3 Faculty of Medicine, Child Health Research Centre, The University of Queensland, Brisbane, QLD, Australia; 4 Department of Population Science, Jatiya Kabi Kazi Nazrul Islam University, Trishal, Mymensingh, Bangladesh; 5 Faculty of Health, Deakin Rural Health, School of Medicine, Deakin University, Warrnambool, Princes Hwy, VIC, Australia; 6 School of Medicine and Public Health, College of Health, Medicine and Wellbeing, University of Newcastle, Callaghan, NSW, Australia; Independent Consultant, UNITED STATES OF AMERICA

## Abstract

**Background:**

Short inter-pregnancy or birth interval is associated with an increased risk of adverse perinatal outcomes. However, some emerging evidence questions this association and there are also inconsistencies among the existing findings. This study aimed to systematically review the evidence regarding the effect of short inter-pregnancy or birth intervals on adverse perinatal outcomes in the Asia-Pacific region.

**Methods:**

A comprehensive search of five databases was conducted targeting studies published between 2000 to 2023. Studies that reported on short inter-pregnancy or birth interval and examined adverse perinatal outcomes, such as low birthweight (LBW) preterm birth (PTB), small for gestational age (SGA), and neonatal mortality were included and appraised for methodological quality using the Joanna Briggs Institute critical appraisal tools. Three reviewers independently screened the studies and performed data extraction. Narrative synthesis and meta-analyses were conducted to summarise the key findings.

**Results:**

A total of 41 studies that fulfilled the inclusion criteria were included. A short-interpregnancy interval was associated with an increased risk of low birthweight (odds ratio [OR] = 1.65; 95%CI:1.39, 1.95), preterm birth (OR = 1.50; 95%CI: 1.35, 1.66), and small for gestational age (OR = 1.24; 95%CI:1.09, 1.41). We also found elevated odds of early neonatal mortality (OR = 1.91; 95%CI: 1.11, 3.29) and neonatal mortality (OR = 1.78; 95%CI: 1.25, 2.55) among women with short birth intervals.

**Conclusion:**

This review indicates that both short inter-pregnancy and birth interval increased the risk of adverse perinatal outcomes. This underscores the importance of advocating for and implementing strategies to promote optimal pregnancy and birth spacing to reduce the occurrence of adverse perinatal outcomes. Reproductive health policies and programs need to be further strengthened and promote access to comprehensive family planning services and increase awareness about the importance of optimal pregnancy and birth spacing.

## Introduction

The time duration between pregnancies or births is considered a significant and modifiable risk factor for adverse pregnancy outcomes [1,2]. The World Health Organization (WHO) recommends a waiting time of at least 24 months from a live birth to a subsequent pregnancy and defines an interval of less than 24 months as a short inter-pregnancy interval [3]. This recommendation has further been extrapolated to the time between two consecutive live births of at least 33 months, considering the nine-month duration of pregnancy [4–8].

Women who conceive within short duration after a previous birth may face higher risks of adverse perinatal outcomes, including preterm birth (PTB) [9], low birthweight (LBW) [10], and small-for-gestational-age (SGA) [10]. Such adverse outcomes can result in both immediate and long-term health problems. For instance, babies born preterm are at increased risk of hospitalisation [11], early mortality [12], and long-term complications, such as developmental delays [13] and chronic health problems in later life [14]. In addition to posing a significant health burden on children, adverse perinatal outcomes can also have negative impacts on psychosocial well-being of families and caregivers as well as on health care resources [15,16].

Despite the WHO-recommended definition, there are inconsistencies between existing studies regarding the definition of short inter-pregnancy or birth interval. Notably, while the WHO defines an interval of <24 months as a short birth-to-pregnancy (inter-pregnancy) interval, this classification is frequently used interchangeably with a short birth interval [17]. Thus, in this review, taking this variation into account, the two concepts were considered together as a “short inter-pregnancy/birth interval” to ensure a comprehensive analysis of associated adverse perinatal outcomes.

An earlier systematic review and meta-analysis reported that an inter-pregnancy interval of <18 months was associated with a higher risk of adverse perinatal outcomes. Specifically, an interval of less than six months was associated with an increased odds of LBW by up to 61%, PTB by 40%, and SGA by up to 26% in the subsequent pregnancy [18]. These findings and others [19,20] were the basis for the 2005 WHO recommendation of a 24 month waiting time before attempting another pregnancy [3]. However, a recent systematic review found inadequate evidence of increased risks of adverse perinatal outcomes, although the review specifically focused on high-income countries and only 28% of the included studies were determined to be of a good quality [21].

The theoretical mechanisms between short inter-pregnancy/birth interval and adverse perinatal and neonatal outcomes relate to intermediating risk factors such as maternal nutritional depletion, folate depletion, horizontal and vertical transmission of infections, cervical insufficiency, sub-optimal breastfeeding, and sibling competition [17,22,23]. For instance, previous studies reported positive association between short interpregnancy/birth interval and maternal anemia during pregnancy [24,25], reduced maternal serum and erythrocyte concentrations of folate [26], under-five morbidities, such as acute respiratory illness and diarrhoea [27,28], and the risk of congenital cytomegalovirus infection [29].

The maternal nutritional depletion hypothesis argues that short inter-pregnancy or birth intervals do not allow women to have sufficient time to fully recover from the preceding pregnancy [30]. The infection transmission (horizontal) hypothesis states that closely spaced pregnancies or births increase the likelihood of infections for the younger sibling, thereby elevating the risk of mortality [31]. Furthermore, sub-optimal breastfeeding and sibling competition is also associated with a higher risk of adverse outcomes, including neonatal mortality, due to breastfeeding-pregnancy overlap and reduced amount of breastmilk for the younger siblings [22,23].

Globally, although most of the existing evidence supports the claim that short inter-pregnancy or birth interval is associated with an increased risk of adverse perinatal outcomes, such as LBW [32,33], PTB [32–34], stillbirth [33,35], SGA [32–34], and neonatal mortality [33,35], other studies, particularly those from high-resource settings, have not found evidence of increased risks [36], with some challenging the universal applicability of the WHO pregnancy interval recommendations [37]. This inconsistency is also commonly seen among the studies conducted in the Asia-Pacific region. For example, while some studies have reported elevated risks for LBW [38–45], PTB [42,43,46–48], stillbirth [49,50], and SGA [42,51,52], others have not reported a significant association between short inter-pregnancy or birth interval and LBW [51–55], PTB [51,52], and SGA [56], leading to inconclusive findings. Such inconsistent findings have raised concerns about the causal relationship between short inter-pregnancy/birth interval and adverse perinatal outcomes. This prompted further evidence synthesis in the area, including updates to the previous systematic review that the WHO recommendation was primarily based on [18]. Contrary to the previous findings, the updated systematic review showed inadequate and inconsistent findings, although the review was limited to studies that were conducted in high-income settings [21].

Given the inconsistencies observed in the existing studies that examined the association between a short inter-pregnancy or birth interval and adverse perinatal outcomes and, most importantly, due to the emerging evidence from high-resource settings that questions the applicability of the WHO recommendation [37], it is critical to revisit the evidence regarding the association between short inter-pregnancy or birth interval and adverse perinatal outcomes and provide summary estimates of the effects to facilitate evidence-informed decision making. The purpose of this study was to systematically summarise the evidence on the association between short inter-pregnancy or birth interval and adverse perinatal outcomes, such as LBW, PTB, and SGA, and provide summary estimates of the effect on each outcome in the Asia-Pacific region.

## Methods

A systematic review and meta-analysis was conducted as a part of a broader review that aimed to map the epidemiology of short birth interval and its impact on maternal and child health outcomes in the Asia-Pacific region. The protocol for the review was registered with PROSPERO (registration number: CRD42023432913). We reported the findings following the Preferred Reporting Items of Systematic Review and Meta-analysis (PRISMA) 2020 statement [57].

### Eligibility criteria

Studies were eligible to be included in this systematic review if they were conducted in the Asia-Pacific region [58,59], written in English, published between September 2000 and May 2023, and reported on adverse perinatal outcomes (e.g. LBW, PTB, stillbirth, SGA, and neonatal mortality) as key outcome variables and considered short inter-pregnancy or birth interval as an exposure variable/covariate. Original research using qualitative, cross-sectional, case-control, cohort, quasi-experimental, non-randomised intervention, or randomised controlled trial (RCT) study designs were considered. Studies were excluded if, (1) data were obtained from multiple countries and where country-specific data could not be disaggregated for Asia-pacific countries, (2) reported short inter-pregnancy or birth intervals in conjunction with birth order (where data for short inter-pregnancy or birth interval could not be disaggregated), (3) considered birth interval as a continuous variable and reported mean or median, instead of considering the variable as a categorical variable and defined short birth interval.

### Information sources

A comprehensive search was conducted across five databases, namely MEDLINE, Scopus, Maternity and Infant Care, Web of Science (WoS), and Cumulative Index to Nursing and Allied Health Literature (CINAHL), to retrieve relevant studies.

### Search strategy

Three authors (DMS, CC, MH) developed a comprehensive search strategy tailored to the requirements of each database. The search strategy was refined in consultation with a librarian from the University of Newcastle and then tested in Medline and Embase. The search was initially conducted in July 2022 and updated in May 2023. Additionally, the reference lists of published review articles were also scrutinised for additional relevant publications. As this systematic review was part of the broader review on short inter-pregnancy or birth intervals in the Asia-Pacific region, we included terms related to short inter-pregnancy or birth interval, prevalence, neonatal and infant health, under-five health, and maternal health to construct comprehensive search strings. We also added the list of the individual countries in the region of interest [58,59] to the search strings, to make the search as comprehensive as possible. The search strategy and results for each of the databases are included in the supplementary material (see S1-S5 Tables in S1 File).

### Selection process

**Fig 1:**
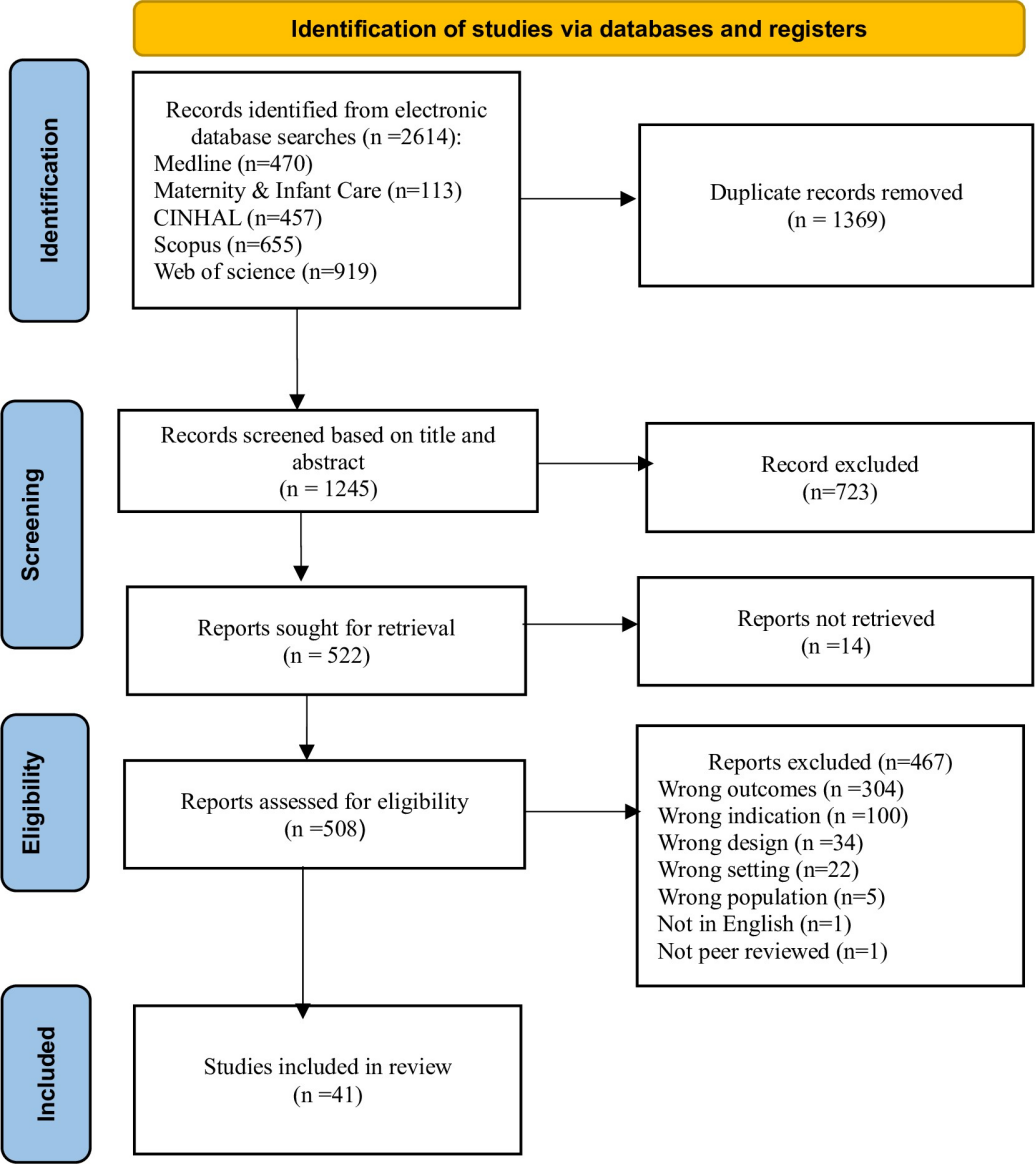
Studies selection process according to PRISMA 2020 statement.

The studies retrieved from each database were exported to the Endnote referencing software version 20.2.1 and checked for duplication. If duplicates were detected, they were recorded and then removed from the Endnote library. The remaining studies were then exported to Covidence for the title and abstract screening and full-text review. Three authors (DMS, CC, MH) independently screened studies by title and abstracts for eligibility. If a study met the eligibility criteria, the entire article (full text) was independently reviewed by three authors (TAH, CC, MH). Where the full text of a study was not accessible, efforts were made to contact the study authors to obtain the full text, if possible. Disagreements during the initial screening and full-text review were resolved through discussion between the reviewers. The reasons for exclusion were documented for the studies excluded in the full-text review (see Fig 1).

### Data collection process and data items

Data for the included studies were initially extracted by TAH and validated by TB and TRF, using a structured Excel spreadsheet. Data were extracted on the following study components: author and year of publication, country, study design, population characteristics, and key findings including the measures of effect and effect size. Furthermore, data on the exposure variable (short inter-pregnancy/birth interval) and outcome variables (LBW, SGA, PTB, stillbirth, neonatal death, and perinatal death) were also extracted.

### Study risk of bias assessment

We used the Joanna Briggs Institute (JBI) critical appraisal tools to assess the risk of bias for the included studies [60]. The JBI critical appraisal tools assess three key aspects of the study: design, conduct, and analysis. Three authors (TAH, TB, TRF) independently rated each study and disagreements were resolved through a discussion. The score for each JBI tool was divided into three categories: high, medium, and low. Studies with higher scores were considered as less prone to methodological bias and categorised as having a low risk of bias. All the studies were included in the results.

### Data synthesis and analysis methods

We employed both qualitative data synthesis and quantitative data analysis to report the findings of this review. The qualitative synthesis was used to report the key findings for each study included along with study parameters (including author and year of publication, country, study design, and population characteristics). To present the summary estimates or the pooled effect sizes of a short inter-pregnancy/birth interval on the measured adverse perinatal outcomes, meta-analyses were conducted as follows: Unadjusted effect sizes with 95% confidence intervals (measured in odds ratios, relative risks, and hazard ratios) were extracted and reported, otherwise manually calculated from raw data, where possible. Where unadjusted effect sizes were not reported or sufficient data were not reported to manually calculate them, adjusted effect sizes were considered.

We used either a fixed-effects or random-effects model to calculate the summary estimates for the overall effect of short inter-pregnancy or birth interval on adverse perinatal outcomes. The extent of heterogeneity was described using I^2^ statistics with a corresponding p-value. When the test for heterogeneity indicated moderate (50%) or high (75%) heterogeneity, the pooled estimates of ORs were computed using the random-effects model. The source of heterogeneity was explored through subgroup and meta-regression analyses based on the pre-specified subgroups such as the classification of short-interpregnancy/birth interval and outcome measured. We assessed a publication bias through visual inspection of Funnel plot asymmetry and Egger’s regression test. We used the Trim and Fill method, where publication bias was detected, to estimate and adjust for potentially missing studies, and re-estimate the effect size. The statistical analysis was performed using Stata software version 15.1 (Stata Corp, College Station, Texas, USA).

### Ethics statements

Ethical approval was not required for this systematic review and meta-analysis as it involved synthesis and analysis of publicly available data from previously published studies.

## Results

### Study inclusion

A total of 2614 studies were identified from five databases on the initial search. Out of the total number of studies identified, 1369 were found to be duplicates and removed. Through initial title and abstract screening, 723 studies were excluded, and 467 studies were excluded after a full-text review. Finally, a total of 41 studies that investigated the association between short birth interval and adverse perinatal outcomes were included in this review (Fig 1).

### Characteristics of the included studies

Of the included studies, about one-quarter of the studies were conducted in India (n = 13 studies) [38–40,49,55,61–66] followed by Pakistan (n = 8) [46,48,67–72] and China (n = 5) [42,51,52,73,74]. The remaining studies were conducted in Bangladesh (n = 3) [45,50,54], Japan (n = 4) [47,75–77], Australia (n = 3) [43,56,78], Afghanistan (n = 2) [53,79], Indonesia (n = 1) [44], Nepal (n = 1) [80], and Papua New Guinea (n = 1) [81]. More than half of the included studies were cross-sectional (n = 23)[38–40,42–45,48–50,52,53,55,61,63,64,68,70,71,79,82–84], one quarter (n = 12)[41,47,51,54,66,67,73,75,78,85–87] were cohort studies, and the remaining six studies were case controls [46,62,72,74,81,88]. Twenty-five studies were facility-based [41–44,46–48,51,61,62,66–68,72–75,78,81,82,84–88], and the remaining 16 studies were community-based studies [38–40,45,49,50,52–55,63,64,70,71,79,83]. Regarding the data collection methods, 29 studies used a predeveloped questionnaire [38–41,44–46,48–55,61–64,67,68,70,71,74,79,81–84], while the remaining 12 utilised record reviews or secondary data [42,43,47,66,72,73,75,78,85–88]. Eleven studies reported two or more adverse perinatal outcomes [42,43,49–52,56,67,68,78,89]. Approximately two-thirds of the included studies reported odd ratios (ORs) as the effect measure [38–40,42,43,45–55,61–63,66,68,70–72,74,75,78,79,81–88] (S6 Table in S1 File). Nearly, 46% of the studies demonstrated a low risk of bias (S7-S9 Tables in S1 File).

### Effect of short inter-pregnancy/birth interval on adverse perinatal outcomes

The studies included in this review investigated short inter-pregnancy or birth intervals in relation to a wide range of adverse perinatal outcomes. Out of 41 studies included, 23 reported on the effects of short inter-pregnancy intervals [36,38,39,41,42,44,46–48,51,52,55,61,65,67,68,73–77,82,85], while 18 studies focused on short birth intervals [5,40,43,45,49,53,54,62–64,66,69–72,79–81]. Of the studies that reported on short inter-pregnancy intervals, 11 used a cut-off point of <6 months to classify short-interpregnancy interval [36,39,42,51,52,67,73–75,85,87]. More than 50% of the studies (n = 10) that provided data on short birth intervals employed a cut-off point of <24 months to classify a short birth interval [40,53,54,62,64,66,79,81,83,84]. Three studies used the WHO extrapolated cut-off point of 33 months to classify a short birth interval [45,50,71]. The following section presents the effects of short inter-pregnancy or birth interval on the reported perinatal outcomes.

### Short inter-pregnancy/birth interval and LBW

The associations between short inter-pregnancy/birth intervals and LBW were investigated in more than half of the studies (n = 22) [38–45,51–56,61,62,67,68,72,78,81,82] included in this review. Out of the 22 studies, 14 specifically provided data on the association between short inter-pregnancy intervals and LBW [38,39,41,42,44,51,52,55,61,67,68,78,82,85], of which eight studies reported an increased risk of LBW among women with short inter-pregnancy interval [38,39,41,42,44,61,67,82] and six did not find sufficient evidence [51,52,55,68,78,85]. Overall, the pooled estimate indicated that the odds of experiencing LBW were 1.65 (OR = 1.65; 95%CI:1.39, 1.95) times higher among women with any inter-pregnancy intervals of <24 months (Table 1). In a stratified analysis, a short inter-pregnancy interval of ≤6 months was found to increase the risk of LBW by 43%.

**Table 1:** Summary estimates of the effects of short inter-pregnancy intervals on adverse perinatal outcomes.

Adverse perinatal outcomes	Number of studies	Summary estimates^a^		Egger bias test p-value	Trim and Fill estimates^b^	
		OR (95% CI)	Heterogeneity Index		missing studies	OR (95% CI)
Fetal growth restriction/intrauterine growth retardation	2	0.95 (0.53–1.68)	0.0	NA	0	0.95 (0.53–1.68)
Low birthweight	12	1.65 (1.39–1.95)	92.8	0.00	0	1.65 (1.39–1.95)
Preterm birth	15	1.50 (1.35–1.66)	78.0	0.00	1	1.44 (1.27–1.61)
Small for gestational age	5	1.24 (1.09–1.41)	90.7	0.00	0	1.24 (1.09–1.41)

Note: Non-short inter-pregnancy interval was considered the reference category

^**a**^Summary estimates were based on random-effects methods except for FGR/IUGR which were based on the fixed-effects model.

^b^The trim-and-fill method simulates studies that are likely to be missing from the literature due to publication or other forms of bias. The trim-and-fill OR estimates what the pooled OR would be if these missing studies were included in the analysis.

A total of eight studies investigated the link between short birth intervals and LBW [40,43,45,53,54,62,72,81]. Of these, six studies reported that short birth interval was an important predictor of LBW [40,43,45,62,72,81] whilst two did not report an increased risk [53,54]. The findings from the meta-analysis showed a non-statistically significant increased risk of LBW within any birth interval of <33 months (Table 2). However, in the sub-group analysis, a short birth interval of <12 months was found to increase the odds of LBW by approximately five-fold (OR = 4.84; 95%CI: 4.42, 5.30), and a short birth interval of <24 months showed an 18% higher risk of LBW, although this increment was not statistically significant (OR = 1.18, 95%CI: 0.67, 20.7).

**Table 2:** Summary estimates of the effects of short birth interval on adverse perinatal outcomes.

Adverse perinatal outcomes	Number of Studies	Summary estimates		Egger bias test p-value	Trim and Fill estimates^a^	
		OR (95% CI)	Heterogeneity Index		missing studies	OR (95% CI)
Low birthweight	7	1.36 (0.62–2.98)	99.4	0.0	0	1.36 (0.62–2.98)
Early neonatal mortality	2	1.91 (1.11–3.29)	57.1	0.12	0	1.91 (1.11–3.29)
Neonatal mortality	6	1.78 (1.25–2.55)	95.2	0.00	0	1.78 (1.25–2.55)
Perinatal mortality	2	1.93 (1.27–2.93)	45.4	0.00	0	1.93 (1.27–2.93)
Preterm birth	3	3.37 (1.95–5.80)	63.5	0.06	0	3.37 (1.95–5.80)
Stillbirth	2	2.44 (2.22–2.69)	0.0	0.516	0	2.44 (2.22–2.69)

Note: Non-short birth interval was considered the reference category.

^**a**^The trim-and-fill method simulates studies that are likely to be missing from the literature due to publication or other forms of bias. The trim-and-fill OR estimates what the pooled OR would be if these missing studies were included in the analysis.

### Short inter-pregnancy/birth interval and PTB

The link between short inter-pregnancy/birth interval and PTB was investigated in 19 studies, of which 16 specifically examined short inter-pregnancy interval [42,46–48,51,52,65,67,68,73–78,85] and three studies reported on short birth intervals [43,66,80]. Of the 16 studies that examined short inter-pregnancy intervals, 10 reported an elevated risk of PTB [42,46–48,65,67,73,75,77,85] following short inter-pregnancy interval, and six did not find evidence of elevated risk [51,52,68,74,76,78]. In addition, all three studies of short birth intervals showed a higher risk of PTB among women with short birth intervals [43,66,80]. The pooled estimate indicated a 50% (OR = 1.50; 95%CI: 1.35, 1.66) higher risk of PTB following an inter-pregnancy interval of <24 months. The subgroup analysis also revealed a similar risk for a short-interpregnancy interval of ≤6 months (OR = 1.50; 95%CI: 1.38, 1.63). Nevertheless, although a higher risk was also observed for an inter-pregnancy interval of <18 months, the risk was found to be not statistically significant (OR = 3.22, 95%C: 0.95, 10.95).

### Short inter-pregnancy/birth interval and intra-uterine growth restriction or SGA

Seven studies investigated either the association between short inter-pregnancy interval and SGA (n = 5) [42,51,52,56,78] or intra-uterine fetal growth restriction (n = 2) [68,76]. In three studies that focused on SGA, short inter-pregnancy interval was found to increase the odds of being SGA [42,51,52], while two studies did not find a statistically significant association [78,85]. Short inter-pregnancy intervals were not found to elevate the odds of fetal growth restriction in both studies [68,76]. The summary estimates showed that the odds being SGA were 1.24 (OR = 1.24; 95%CI:1.09,1.41) times higher among babies born within a short inter-pregnancy interval of ≤6 months. No studies reported on the association between short birth interval and SGA.

## Short inter-pregnancy/birth interval and perinatal deaths

The relationships between short birth intervals and perinatal deaths or its individual components (stillbirth and neonatal death) were investigated in seven studies [49,50,63,64,69,70,79]. Of these studies, neonatal mortality was reported in all studies [49,50,63,64,69,70,79], stillbirth in two studies [49,50], and perinatal mortality was specifically reported in two studies [50,69]. The results of the individual studies indicated that short birth interval was significantly associated with increased risks of neonatal deaths in six studies [49,50,63,69,70,79] whilst one study reported a reduced risk [64]. The summary estimates showed that short birth intervals were significantly associated with early neonatal mortality (OR = 1.91; 95%CI: 1.11, 3.29), neonatal mortality (OR = 1.78; 95%CI: 1.25, 2.55), stillbirth (OR = 2.44; 95%CI: 2.22, 2.69), and perinatal mortality (OR = 1.93; 95%CI1: 1.27, 2.93). The stratified analysis by the classification used revealed a 35% and 64% higher risk of neonatal mortality for a short birth interval of <12 months and <24 months, respectively. In addition, although the findings of the individual studies showed increased risks of experiencing stillbirths [49,50] and perinatal deaths [50,69] among women with a history of a short birth interval, there were no sufficient studies to provide stratified summary estimates for these outcomes. Furthermore, there were no studies that provided data on the association between short interpregnancy interval and perinatal deaths.

## Discussion

This systematic review and meta-analysis comprehensively synthesised evidence on the effects of short inter-pregnancy or birth intervals on adverse perinatal outcomes in the Asia-Pacific region. The findings indicated that while a short inter-pregnancy increased the likelihood of experiencing LBW, PTB, and SGA, a short birth interval was found to increase the odds of neonatal mortality (including early neonatal mortality), stillbirth, and perinatal mortality.

Our findings revealed a 43% increased risk of LBW following a short inter-pregnancy interval. This is consistent with a previous systematic review and meta-analysis, which included studies both from both low and high-resource settings globally, and revealed increased odds of LBW among women with a short inter-pregnancy interval [18]. Specifically, the previous review reported an odds ratio of 1.61 for inter-pregnancy intervals of less than six months compared to inter-pregnancy interval of 18–23 months. Therefore, our findings not only corroborate these findings but also provide updated estimates, underscoring the persistent and significant impact of short inter-pregnancy intervals on LBW across diverse populations. The link between a short inter-pregnancy interval and LBW could be attributed to a range of underlying mechanisms, including maternal nutritional depletion, increased physiological demands on the maternal body, stress and emotional strain [90]. Short intervals between pregnancies do not provide sufficient time for maternal nutrient replenishment [91], leading to inadequate maternal nutrition which, in turn, affects fetal growth and development [30,92]. Furthermore, closely spaced pregnancies might also shorten the time for identifying and addressing preconception risk factors, such as maternal mental health conditions which tend to elevate the risk of pregnancy complications, including LBW [93].

In line with earlier systematic reviews conducted globally [18] or in high-resource settings [21], our review found that a short inter-pregnancy interval significantly increased the odds of experiencing PTB. While the previous review conducted in high-resource settings reported an odds ratio of 1.20 for inter-pregnancy interval of less than 6 months [21], our updated analysis indicated a slightly higher odds ratio of 1.50. This difference could be attributed to the differences in sociodemographic characteristics and health service delivery as our analysis mainly included data from low- and middle-income countries where the effect of short interpregnancy interval may be more pronounced [94].

A short inter-pregnancy interval may lead to various interconnected factors that increase the risk of PTB [17]. One of such intermediating factors might be insufficient recovery time for the uterus and cervix which might lead to overactivity of uterine muscles and premature cervical dilatation that triggers premature contractions and premature membrane rupture [95,96]. Like that of LBW, a short birth interval could also contribute to maternal nutritional depletion and increased stress, causing hormonal imbalances that provoke preterm contractions. Maternal nutritional depletion may also lead to compromised immunity, increasing susceptibility to infections and inflammation that could trigger preterm labour. Despite this, some of studies have not found evidence of elevated risk for PTB among women with short inter-pregnancy intervals [52,78], highlighting the need for further investigation.

We also found an increased risk of SGA in pregnancies that occurred following short inter-pregnancy intervals. This finding is in line with most of the previously conducted studies which indicated an increased risk of SGA among women with short inter-pregnancy intervals [97,98]. For example, a large population-based study conducted in Canada showed an elevated risk of SGA among women with inter-pregnancy interval of less than 6 months compared to those with 18 months inter-pregnancy interval [99]. The possible mechanisms by which short inter-pregnancy intervals are linked to SGA may include maternal nutritional (including folate) depletion and vertical transmission of infections [17,91]. The maternal nutritional depletion hypothesis states that short intervals between pregnancies could deteriorate the mother’s nutritional well-being, due to insufficient time for the mother to recover from the physiological stresses of the prior pregnancy before being exposed to the stresses of the subsequent pregnancy [100]. Consequently, the mother’s nutritional status at conception might be compromised, which negatively affects fetal growth and development, resulting in SGA. Maternal infections are associated with an increased risk of adverse perinatal outcomes, such as fetal growth restriction [101], and it has been hypothesised that the risk of vertical infection transmission from the mother to the fetus could be increased among women with short inter-pregnancy intervals [17].

The current review showed increased odds of experiencing perinatal death (including stillbirth and neonatal deaths) among women with short birth intervals, congruent to the findings reported in previous study. For example, a systematic review and meta-analysis that included studies from low and middle income countries, including studies conducted in countries of the Asia-Pacific region, reported increased risks of perinatal mortality, neonatal mortality, early neonatal, and post neonatal mortality following short birth intervals [6].

The possible causal mechanisms for this linkage could be through intermediating factors such as the transmission of infections (vertical and among siblings), sibling competition, and suboptimal lactation related to breastfeeding–pregnancy overlap [17]. In addition to vertical mother to fetus transmission of infections, closely spaced births may place the younger sibling at a higher risk of infections and, thereby, of mortality, due to the possibility of horizontal transmission of infections between siblings. Earlier literature also documented that younger children contracting secondary infections from their older siblings tend to have higher case fatality rates for some communicable childhood diseases, such as measles [102,103].

Although the sibling competition pathway has been highly cited for the linkage between short inter-pregnancy or birth interval and overall adverse child outcomes [104,105], it was found to be inconclusive for early mortality, particularly neonatal mortality, when the survival status of the preceding child was considered into account [17]. The survival status of the preceding sibling has a potential confounding effect to influence the duration of inter-pregnancy or birth interval. This is because when the preceding sibling dies, women tend to have shorter intervals for the next pregnancy due to involuntary cessation of breastfeeding and or a desire to replace the deceased child [17].

Accounting for all other important factors, subsequent children born to women whose preceding child dies are expected to have a lower risk of mortality as compared to those born to women whose preceding child survives, if the sibling competition hypothesis holds true for all forms of child mortality. The earlier systematic review of causal mechanisms, however, reported conflicting evidence supporting the sibling competition hypothesis, particularly for neonatal mortality [17]. This highlights the need for further primary studies that investigate the association between the preceding inter-pregnancy and birth interval and neonatal mortality, particularly according to the survival status of the preceding child.

Given the profound impact of short inter-pregnancy or birth intervals on perinatal outcomes, healthcare providers should educate women and families about the importance of sufficient time between pregnancies or births for adequate maternal recovery and proper prenatal care. In addition to this, it is crucial to enhance the availability and accessibility of effective contraception to help women and couples actively plan and control the timing of their pregnancies, thereby reducing the risk of adverse perinatal outcomes. Policies and programs supporting family planning and reproductive health services need to be further strengthened in promoting healthy birth spacing and ensuring that individuals have access to the necessary contraception methods to support their reproductive choices while safeguarding maternal and infant health.

Equally important, there is a need to use consistent definition for short inter-pregnancy as well as short birth interval to generate data for evidence-informed decisions and policymaking. Although the WHO recommends a waiting time of at least 24 months from a live birth to a subsequent pregnancy and consequently defines an interval less than 24 months as a short birth-to-pregnancy interval, there is a poor adherence to this recommendation, particularly among studies conducted in low-and middle-income countries [6,106]. It is also becoming a common practice to extrapolate this WHO recommendation to measure a birth-to-birth interval, by accounting for the duration (nine months) of the subsequent pregnancy [5,8,107,108]. Although this method may be more intuitive for collecting the data, particularly where there is a lack of precise birth registration, it is of some limitations. This is because the method assumes that pregnancy ends at nine months, which may not always be the case, particularly for preterm and some stillbirths. Consequently, women with a preterm birth would systematically have short birth intervals and this would potentially lead to the issue of reverse causation when investigating short birth intervals and adverse perinatal outcomes [21,109]. Therefore, while this simplified method may provide valuable insights into birth spacing, researchers should exercise caution when applying it. Furthermore, whenever possible, the WHO-recommended definition of birth-to-pregnancy interval (also known as interpregnancy interval) needs to be followed for consistency.

Future research should investigate the underlying mechanisms and contextual factors that influence the increased risk of adverse perinatal outcomes associated with short inter-pregnancy and birth intervals. Comparative studies using consistent definitions of these intervals are also needed to determine if their effects vary across different contexts, such as socioeconomic settings and healthcare systems. Understanding these contextual differences will enhance the applicability and effectiveness of targeted interventions aimed at optimizing inter-pregnancy and birth intervals, ultimately reducing adverse perinatal outcomes.

### Strengths and limitations of the study

This is the first study to comprehensively present both the effects of short inter-pregnancy intervals and short birth intervals on adverse perinatal outcomes. We conducted a comprehensive search of five different databases and included studies conducted over an extended period (2000–2023). We considered the studies reported on short interpregnancy intervals and birth intervals and conducted several subgroup analyses based on the classification of short inter-pregnancy and birth intervals. By doing so, we were able to provide summary estimates for various strata of short inter-pregnancy and birth intervals on a wide range of adverse perinatal outcomes. Although we were able to provide the pooled estimates of short inter-pregnancy and birth intervals on the adverse perinatal outcomes, there were no sufficient studies to present stratified pooled estimates for some outcomes, based on the classification used, warranting further studies. While efforts were made to understand the sources of heterogeneity, the results provided no plausible explanation. This warrants caution when interpreting the findings as the observed heterogeneity could stem from complex interactions between biological, socio-economic, and healthcare system factors.

There were also limited studies that strictly followed the WHO-recommended definition of a short birth-to-pregnancy interval (<24 months), particularly in the countries of the Asia-Pacific region. Another limitation of this systematic review and meta-analysis is the quality of included studies. Nearly half of the studies were assessed to be of medium or low quality. This variability in study quality may affect the precision of the pooled estimate, warranting caution when interpreting the findings.

## Conclusion

This study showed that both short inter-pregnancy and birth intervals were associated with an increased likelihood of adverse perinatal outcomes such as LBW, PTB, SGA, and neonatal mortality. This underscores the importance of advocating for and implementing strategies to promote optimal pregnancy or birth spacing to reduce the occurrence of adverse perinatal outcomes. Specific strategies could include increasing access to comprehensive family planning services, enhancing public awareness campaigns about the benefits of optimal birth spacing, and integrating birth spacing counselling into routine maternal and child healthcare.

## Supporting information

S1 File Supplementary materials for the effects of short inter-pregnancy/birth interval on adverse perinatal outcomes in Asia-Pacific Region: A systematic review and meta-analysis. (DOCX)

## References

[1] Zhu BP. Effect of interpregnancy interval on birth outcomes: findings from three recent US studies. International Journal of Gynecology & Obstetrics. 2005;89:S25–S33. doi: 10.1016/j.ijgo.2004.08.002 15820365

[2] Shachar BZ, Lyell DJ. Interpregnancy interval and obstetrical complications. Obstetrical & gynecological survey. 2012;67(9):584–96. doi: 10.1097/OGX.0b013e31826b2c3e 22990461

[3] World Health Organization. Report of a WHO technical consultation on birth spacing: Geneva, Switzerland 13–15 June 2005. World Health Organization, 2007.

[4] Exavery A, Mrema S, Shamte A, Bietsch K, Mosha D, Mbaruku G, et al. Levels and correlates of non-adherence to WHO recommended inter-birth intervals in Rufiji, Tanzania. BMC pregnancy and childbirth. 2012;12:1–8. 23237623 10.1186/1471-2393-12-152 PMC3573999

[5] De Jonge HC, Azad K, Seward N, Kuddus A, Shaha S, Beard J, et al. Determinants and consequences of short birth interval in rural Bangladesh: a cross-sectional study. BMC pregnancy and childbirth. 2014;14(1):1–7. doi: 10.1186/s12884-014-0427-6 25539669 PMC4314752

[6] Islam MZ, Billah A, Islam MM, Rahman M, Khan N. Negative effects of short birth interval on child mortality in low-and middle-income countries: a systematic review and meta-analysis. Journal of Global Health. 2022;12. doi: 10.7189/jogh.12.04070 36057919 PMC9441110

[7] Islam MZ, Islam MM, Rahman MM, Khan MN. Prevalence and risk factors of short birth interval in Bangladesh: Evidence from the linked data of population and health facility survey. PLOS Global Public Health. 2022;2(4):e0000288. doi: 10.1371/journal.pgph.0000288 36962161 PMC10021594

[8] Shifti DM, Chojenta C, Holliday E, Loxton D. Effects of short birth interval on neonatal, infant and under-five child mortality in Ethiopia: a nationally representative observational study using inverse probability of treatment weighting. BMJ open. 2021;11(8):e047892. doi: 10.1136/bmjopen-2020-047892 34408041 PMC8375759

[9] Shachar BZ, Mayo JA, Lyell DJ, Baer R, Jeliffe‐Pawlowski L, Stevenson D, et al. Interpregnancy interval after live birth or pregnancy termination and estimated risk of preterm birth: a retrospective cohort study. BJOG: An International Journal of Obstetrics & Gynaecology. 2016;123(12):2009–17. doi: 10.1111/1471-0528.14165 27405702

[10] Xu T, Miao H, Chen Y, Luo L, Guo P, Zhu Y. Association of interpregnancy interval with adverse birth outcomes. JAMA Network Open. 2022;5(6):e2216658–e. doi: 10.1001/jamanetworkopen.2022.16658 35696164 PMC9194661

[11] Do CHT, Børresen ML, Pedersen FK, Geskus RB, Kruse AY. Rates of rehospitalisation in the first 2 years among preterm infants discharged from the NICU of a tertiary children hospital in Vietnam: a follow-up study. BMJ open. 2020;10(10):e036484. doi: 10.1136/bmjopen-2019-036484 33020086 PMC7537446

[12] Kannaujiya AK, Kumar K, Upadhyay AK, McDougal L, Raj A, James K, et al. Effect of preterm birth on early neonatal, late neonatal, and postneonatal mortality in India. PLOS global public health. 2022;2(6):e0000205. doi: 10.1371/journal.pgph.0000205 36962696 PMC10021707

[13] Cheong JL, Doyle LW, Burnett AC, Lee KJ, Walsh JM, Potter CR, et al. Association between moderate and late preterm birth and neurodevelopment and social-emotional development at age 2 years. JAMA pediatrics. 2017;171(4):e164805–e. doi: 10.1001/jamapediatrics.2016.4805 28152144

[14] Chehade H, Simeoni U, Guignard J-P, Boubred F. Preterm birth: long term cardiovascular and renal consequences. Current pediatric reviews. 2018;14(4):219–26. doi: 10.2174/1573396314666180813121652 30101715 PMC6416185

[15] Vigod SN, Villegas L, Dennis CL, Ross LE. Prevalence and risk factors for postpartum depression among women with preterm and low‐birth‐weight infants: a systematic review. BJOG: An International Journal of Obstetrics & Gynaecology. 2010;117(5):540–50. doi: 10.1111/j.1471-0528.2009.02493.x 20121831

[16] Petrou S, Yiu HH, Kwon J. Economic consequences of preterm birth: a systematic review of the recent literature (2009–2017). Archives of disease in childhood. 2019;104(5):456–65. doi: 10.1136/archdischild-2018-315778 30413489

[17] Conde‐Agudelo A, Rosas‐Bermudez A, Castaño F, Norton MH. Effects of birth spacing on maternal, perinatal, infant, and child health: a systematic review of causal mechanisms. Studies in family planning. 2012;43(2):93–114. doi: 10.1111/j.1728-4465.2012.00308.x 23175949

[18] Conde-Agudelo A, Rosas-Bermúdez A, Kafury-Goeta AC. Birth spacing and risk of adverse perinatal outcomes: a meta-analysis. Jama. 2006;295(15):1809–23. doi: 10.1001/jama.295.15.1809 16622143

[19] DaVanzo J, Hale L, Razzaque A, Rahman M. The effects of pregnancy spacing on infant and child mortality in Matlab, Bangladesh: how they vary by the type of pregnancy outcome that began the interval. Population studies. 2008;62(2):131–54. doi: 10.1080/00324720802022089 18587691

[20] Rutstein SO. Effects of preceding birth intervals on neonatal, infant and under‐five years mortality and nutritional status in developing countries: evidence from the demographic and health surveys. International Journal of Gynecology & Obstetrics. 2005;89:S7–S24. doi: 10.1016/j.ijgo.2004.11.012 15820369

[21] Ahrens KA, Nelson H, Stidd RL, Moskosky S, Hutcheon JA. Short interpregnancy intervals and adverse perinatal outcomes in high‐resource settings: an updated systematic review. Paediatric and perinatal epidemiology. 2019;33(1):O25–O47. doi: 10.1111/ppe.12503 30353935 PMC7379643

[22] Marquis GS, Penny ME, Zimmer JP, Díaz JM, Marín RM. An overlap of breastfeeding during late pregnancy is associated with subsequent changes in colostrum composition and morbidity rates among Peruvian infants and their mothers. The Journal of nutrition. 2003;133(8):2585–91. doi: 10.1093/jn/133.8.2585 12888642 PMC2798150

[23] Marquis GS, Penny ME, Diaz JM, Marín RM. Postpartum consequences of an overlap of breastfeeding and pregnancy: reduced breast milk intake and growth during early infancy. Pediatrics. 2002;109(4):e56–e. doi: 10.1542/peds.109.4.e56 11927729 PMC2782541

[24] Conde-Agudelo A, Belizán JM. Maternal morbidity and mortality associated with interpregnancy interval: cross sectional study. Bmj. 2000;321(7271):1255–9. doi: 10.1136/bmj.321.7271.1255 11082085 PMC27528

[25] Dairo MD, Lawoyin TO. Socio-demographic determinants of anaemia in pregnancy at primary care level: a study in urban and rural Oyo State, Nigeria. African journal of medicine and medical sciences. 2004;33(3):213–7. 15819466

[26] Megahed MA, Taher I. Folate and homocysteine levels in pregnancy. British journal of biomedical science. 2004;61(2):84–7. doi: 10.1080/09674845.2004.11732649 15250671

[27] Fagbamigbe AF, Adebola OG, Dukhi N, Fagbamigbe OS, Uthman OA. Exploring the socio-economic determinants of educational inequalities in diarrhoea among under-five children in low-and middle-income countries: a Fairlie decomposition analysis. Archives of Public Health. 2021;79(1):114. doi: 10.1186/s13690-021-00639-8 34167581 PMC8223382

[28] Rahman M, Hosen A, Khan MA. Association between maternal high-risk fertility behavior and childhood morbidity in Bangladesh: a nationally representative cross-sectional survey. The American journal of tropical medicine and hygiene. 2019;101(4):929. doi: 10.4269/ajtmh.19-0221 31333165 PMC6779183

[29] Fowler KB, Stagno S, Pass RF. Interval between births and risk of congenital cytomegalovirus infection. Clinical infectious diseases. 2004;38(7):1035–7. doi: 10.1086/382533 15034839

[30] Winkvist A, Rasmussen KM, Habicht J-P. A new definition of maternal depletion syndrome. American journal of public health. 1992;82(5):691–4. doi: 10.2105/ajph.82.5.691 1566948 PMC1694126

[31] Boerma JT, Bicego GT. Preceding birth intervals and child survival: searching for pathways of influence. Studies in family planning. 1992;23(4):243–56. 1412597

[32] Gurmu L, Wakgari N, Kolola T, Danusa KT. Effect of short inter-pregnancy interval on perinatal outcomes among pregnant women in North-west Ethiopia: A prospective cohort study. Frontiers in Public Health. 2022;10:953481. doi: 10.3389/fpubh.2022.953481 36003632 PMC9393389

[33] Conde-Agudelo A, Belizán JM, Norton MH, Rosas-Bermúdez A. Effect of the interpregnancy interval on perinatal outcomes in Latin America. Obstetrics & Gynecology. 2005;106(2):359–66. 16055588 10.1097/01.AOG.0000171118.79529.a3

[34] Grisaru-Granovsky S, Gordon E-S, Haklai Z, Samueloff A, Schimmel MM. Effect of interpregnancy interval on adverse perinatal outcomes—a national study. Contraception. 2009;80(6):512–8. doi: 10.1016/j.contraception.2009.06.006 19913144

[35] Bauserman M, Nowak K, Nolen TL, Patterson J, Lokangaka A, Tshefu A, et al. The relationship between birth intervals and adverse maternal and neonatal outcomes in six low and lower-middle income countries. Reproductive health. 2020;17(2):1–10. doi: 10.1186/s12978-020-01008-4 33256784 PMC7708104

[36] Ball SJ, Pereira G, Jacoby P, De Klerk N, Stanley FJ. Re-evaluation of link between interpregnancy interval and adverse birth outcomes: retrospective cohort study matching two intervals per mother. Bmj. 2014;349. doi: 10.1136/bmj.g4333 25056260 PMC4137882

[37] Tessema GA, Marinovich ML, Håberg SE, Gissler M, Mayo JA, Nassar N, et al. Interpregnancy intervals and adverse birth outcomes in high-income countries: An international cohort study. PLoS One. 2021;16(7):e0255000. doi: 10.1371/journal.pone.0255000 34280228 PMC8289039

[38] Borah M, Agarwalla R. Maternal and socio-demographic determinants of low birth weight (LBW): A community-based study in a rural block of Assam. Journal of Postgraduate Medicine. 2016;62(3):178–81. doi: 10.4103/0022-3859.184275 Language: English. Entry Date: . Revision Date: 20180326. Publication Type: Article. 27320953 PMC4970345

[39] Kannaujiya AK, Kumar K, Upadhyay AK, McDougal L, Raj A, Singh A. Short interpregnancy interval and low birth weight births in India: Evidence from National Family Health Survey 2015–16. SSM Popul Health. 2020;12:100700. doi: 10.1016/j.ssmph.2020.100700 . 33304985 PMC7710655

[40] Metgud CS, Naik VA, Mallapur MD. Factors affecting birth weight of a newborn—a community based study in rural Karnataka, India. PLoS ONE. 2012;7(7):e40040. doi: 10.1371/journal.pone.0040040 . 22792210 PMC3390317

[41] Negi KS, Kandpal SD, Kukreti M. Epidemiological factors affecting low birth weight. JK Science. 2006;8(1):31–4.

[42] Xu T, Miao H, Chen Y, Luo L, Guo P, Zhu Y. Association of Interpregnancy Interval With Adverse Birth Outcomes. JAMA netw. 2022;5(6):e2216658. doi: 10.1001/jamanetworkopen.2022.16658 . 35696164 PMC9194661

[43] Mohsin M, Jalaludin B. Influence of previous pregnancy outcomes and continued smoking on subsequent pregnancy outcomes: an exploratory study in Australia. Bjog. 2008;115(11):1428–35. doi: 10.1111/j.1471-0528.2008.01864.x . 18700893

[44] Ismah Z, Tjekyan S, Novrikasari. Incident of Low Fetal Weight in Relation to the Interval Between Pregnancies. 2018. p. 6677–80.

[45] Chowdhury M, Dibley MJ, Alam A, Huda TM, Raynes-Greenow C. Household Food Security and Birth Size of Infants: Analysis of the Bangladesh Demographic and Health Survey 2011. Curr. 2018;2(3):nzy003. doi: 10.1093/cdn/nzy003 . 30019026 PMC6041808

[46] Hanif A, Ashraf T, Pervaiz MK, Güler N. Maternal, fetal and neonatal risk factors for preterm birth in parity > 1. Pakistan Paediatric Journal. 2019;43(4):270–7.

[47] Yamashita M, Hayashi S, Endo M, Okuno K, Fukui O, Mimura K, et al. Incidence and risk factors for recurrent spontaneous preterm birth: A retrospective cohort study in Japan. Journal of Obstetrics & Gynaecology Research. 2015;41(11):1708–14. doi: 10.1111/jog.12786 Language: English. Entry Date: . Revision Date: 20180810. Publication Type: Article. 26311118

[48] Fatima M, Naz U, Hira AK, Habib A, Kazi PS, Majeed H. Association Between Pre-Term Labour and Inter Pregnancy Interval. PAKISTAN JOURNAL OF MEDICAL & HEALTH SCIENCES. 2021;15(10):3137–9. doi: 10.53350/pjmhs2115103137 WOS:000730429300083.

[49] Williams EK, Hossain MB, Sharma RK, Kumar V, Pandey CM, Baqui AH. Birth interval and risk of stillbirth or neonatal death: findings from rural north India. J Trop Pediatr. 2008;54(5):321–7. doi: 10.1093/tropej/fmn027 . 18443009

[50] de Jonge HCC, Azad K, Seward N, Kuddus A, Shaha S, Beard J, et al. Determinants and consequences of short birth interval in rural Bangladesh: a cross-sectional study. BMC Pregnancy Childbirth. 2014;14:427. doi: 10.1186/s12884-014-0427-6 . 25539669 PMC4314752

[51] Huo X-X, Gao E-S, Cheng Y-M, Luo L, Liang H, Huang G-Y, et al. Effect of interpregnancy interval after a mifepristone-induced abortion on neonatal outcomes in subsequent pregnancy. Contraception. 2013;87(1):38–44. doi: 10.1016/j.contraception.2012.08.043 . 23040132

[52] Shi G, Zhang B, Kang Y, Dang S, Yan H. Association of Short and Long Interpregnancy Intervals with Adverse Birth Outcomes: Evidence from a Cross-Sectional Study in Northwest China. Int J Gen Med. 2021;14:2871–81. doi: 10.2147/IJGM.S315827 . 34234517 PMC8254096

[53] Das Gupta R, Swasey K, Burrowes V, Hashan MR, Al Kibria GM. Factors associated with low birth weight in Afghanistan: a cross-sectional analysis of the demographic and health survey 2015. BMJ Open. 2019;9(5):e025715. doi: 10.1136/bmjopen-2018-025715 . 31092648 PMC6530387

[54] Hosain GM, Chatterjee N, Begum A, Saha SC. Factors associated with low birthweight in rural Bangladesh. J Trop Pediatr. 2006;52(2):87–91. doi: 10.1093/tropej/fmi066 . 16014761

[55] Kader M, Perera NKPP. Socio-economic and nutritional determinants of low birth weight in India. N A J Med Sci (Hamilt). 2014;6(7):302–8. doi: 10.4103/1947-2714.136902 . 25077077 PMC4114006

[56] Regan AK, Gissler M, Magnus MC, Haberg SE, Ball S, Malacova E, et al. Association between interpregnancy interval and adverse birth outcomes in women with a previous stillbirth: an international cohort study. Lancet. 2019;393(10180):1527–35. doi: 10.1016/S0140-6736(18)32266-9 . 30827781

[57] Page MJ, McKenzie JE, Bossuyt PM, Boutron I, Hoffmann TC, Mulrow CD, et al. The PRISMA 2020 statement: an updated guideline for reporting systematic reviews. International journal of surgery. 2021;88:105906. doi: 10.1016/j.ijsu.2021.105906 33789826

[58] The World Bank. East Asia & Pacific [cited 2022 30 June]. Available from: https://data.worldbank.org/country/Z4 accessed 30 June 2022.

[59] The World Bank. South Asia [cited 2022 30 June]. Available from: https://data.worldbank.org/country/8S accessed 30 June 2022.

[60] Moola S, Munn Z, Tufanaru C, Aromataris E, Sears K, Sfetcu R, et al. Chapter 7: Systematic reviews of etiology and risk. Joanna briggs institute reviewer’s manual The Joanna Briggs Institute. 2017;5:217–69.

[61] Kaur S, Upadhyay AK, Srivastava DK, Srivastava R, Pandey ON. Maternal correlates of birth weight of newborn: A hospital based study. Indian Journal of Community Health. 2014;26(2):187–91. Language: English. Entry Date: 20140708. Revision Date: 20190723. Publication Type: Article.

[62] Nagargoje MM, Chaudhary SS, Deshmukh JS, Gupta SC, Misra SK. A case control study for risk factors of low birth weight in Nagpur city of Maharashtra. INDIAN JOURNAL OF COMMUNITY HEALTH. 2011;23(1):4–7. WOS:000216632800002.

[63] Agrawal S, Agrawal PK, Williams EK, Darmstadt GL, Kumar V, Kiran U, et al. Rural community-based maternal and newborn interventions on prevention of neonatal morality. Nova Science Publishers, Inc.; 2016. p. 17–34.

[64] Patel KK, Kumar M. Differential and Determinants of Neonatal Mortality: A Comparative Study in Northern and Southern Regions of India. Indian J. 2021;46(3):405–10. doi: 10.4103/ijcm.IJCM_425_20 . 34759476 PMC8575228

[65] Reddy KM, Ravula SR, Palakollu S, Betha K. Prevalence of preterm birth and perinatal outcome: A rural tertiary teaching hospital-based study. J Fam Med Prim Care. 2022;11(7):3909–14. doi: 10.4103/jfmpc.jfmpc_1440_21 WOS:000892385900083. 36387651 PMC9648210

[66] Bera M, Chaudhury N, Samanta S. Interpregnancy Interval Effect on Perinatal Outcome- A Prospective Observational Study. J Clin Diagn Res. 2023;17(4):QC1–QC4. doi: 10.7860/jcdr/2023/57940.17706 WOS:000972070100003.

[67] Arshad A, Javaid MK, Rehman A. Comparison of Perinatal Outcome (Low Birth Weight, Preterm Delivery) in Women with < 6 Month Versus 12–17 Months of Interpregnancy Birth Interval. PAKISTAN JOURNAL OF MEDICAL & HEALTH SCIENCES. 2021;15(10):2742–5. doi: 10.53350/pjmhs2115102742 WOS:000730416300084.

[68] Latif L, Iqbal UJ. Fetomaternal Outcomes of Short Inter-pregnancy Interval. PAKISTAN JOURNAL OF MEDICAL & HEALTH SCIENCES. 2019;13(2):424–6. WOS:000483412400072.

[69] Murtaza K, Saleem Z, Jabeen S, Alzahrani AK, Kizilbash N, Soofi SB, et al. Impact of interpregnancy intervals on perinatal and neonatal outcomes in a multiethnic Pakistani population. J Trop Pediatr. 2022;68(6):06. doi: 10.1093/tropej/fmac088 . 36228309

[70] Hussain R. Risk factors for neonatal mortality in low-income population subgroups in Karachi, Pakistan. Community Genet. 2002;5(4):249–56. doi: 10.1159/000066688 . 14960879

[71] Asif MF, Meherali S, Abid G, Khan MS, Lassi ZS. Predictors of Child’s Health in Pakistan and the Moderating Role of Birth Spacing. Int J Environ Res Public Health. 2022;19(3):03. doi: 10.3390/ijerph19031759 . 35162782 PMC8835252

[72] Memon Y, Sheikh S, Memon A. MATERNAL RISK FACTORS AFFECTING BIRTH WEIGHT OF NEWBORN. JOURNAL OF THE LIAQUAT UNIVERSITY OF MEDICAL AND HEALTH SCIENCES. 2005;4(3):94–9. WOS:000216854200003.

[73] Zhang L, Shen S, He J, Chan F, Lu J, Li W, et al. Effect of Interpregnancy Interval on Adverse Perinatal Outcomes in Southern China: A Retrospective Cohort Study, 2000–2015. Paediatr Perinat Epidemiol. 2018;32(2):131–40. doi: 10.1111/ppe.12432 . 29293278

[74] Zhang Y-P, Liu X-H, Gao S-H, Wang J-M, Gu Y-S, Zhang J-Y, et al. Risk factors for preterm birth in five Maternal and Child Health hospitals in Beijing. PLoS ONE. 2012;7(12):e52780. doi: 10.1371/journal.pone.0052780 . 23300774 PMC3531336

[75] Tanigawa K, Ikehara S, Cui M, Kawanishi Y, Kimura T, Ueda K, et al. Association between interpregnancy interval and risk of preterm birth and its modification by folate intake: the Japan Environment and Children’s Study. J Epidemiol. 2021;22:22. doi: 10.2188/jea.JE20210031 . 34024875 PMC9909173

[76] Nakamura Y, Tsuda H, Masahashi Y, Nakamura T, Suzuki M, Fukuhara N, et al. Impact of the interpregnancy interval after cesarean delivery on subsequent perinatal risks: a retrospective study. Arch Gynecol Obstet. 2022:7. doi: 10.1007/s00404-022-06651-9 WOS:000842466300001. 35984489

[77] Tanigawa K, Ikehara S, Cui M, Kawanishi Y, Kimura T, Ueda K, et al. Association Between Interpregnancy Interval and Risk of Preterm Birth and Its Modification by Folate Intake: The Japan Environment and Children’s Study. J Epidemiol. 2023;33(3):113–9. doi: 10.2188/jea.JE20210031 . 34024875 PMC9909173

[78] Ball SJ, Pereira G, Jacoby P, de Klerk N, Stanley FJ. Re-evaluation of link between interpregnancy interval and adverse birth outcomes: retrospective cohort study matching two intervals per mother. Bmj. 2014;349:g4333. doi: 10.1136/bmj.g4333 . 25056260 PMC4137882

[79] Kibria GMA, Burrowes V, Choudhury A, Sharmeen A, Ghosh S, Mahmud A, et al. Determinants of early neonatal mortality in Afghanistan: an analysis of the Demographic and Health Survey 2015. Global health. 2018;14(1):47. doi: 10.1186/s12992-018-0363-8 . 29743085 PMC5944060

[80] Acharya D, Gautam S, Poder TG, Lewin A, Gaussen A, Lee K, et al. Maternal and dietary behavior-related factors associated with preterm birth in Southeastern Terai, Nepal: A cross sectional study. Front. 2022;10:946657. doi: 10.3389/fpubh.2022.946657 . 36187702 PMC9521356

[81] Peters HR, Vince JD, Friesen H. Low birthweight at a Papua New Guinea highlands hospital. J Trop Pediatr. 2001;47(1):17–23. doi: 10.1093/tropej/47.1.17 . 11245345

[82] Kumar S, Dabral M, Jaiswal K, Singh CM. A STUDY ON MATERNAL FACTORS AND PREGNANCY OUTCOME IN MEDICAL COLLEGE HOSPITAL OF JHANSI CITY. INDIAN JOURNAL OF COMMUNITY HEALTH. 2005;17(1–2):5–9. WOS:000216588700002.

[83] Acharya D, Gautam S, Poder TG, Lewin A, Gaussen A, Lee K, et al. Maternal and dietary behavior-related factors associated with preterm birth in Southeastern Terai, Nepal: A cross sectional study. Frontiers in Public Health. 2022;10:946657. doi: 10.3389/fpubh.2022.946657 36187702 PMC9521356

[84] Murtaza K, Saleem Z, Jabeen S, Alzahrani AK, Kizilbash N, Soofi SB, et al. Impact of interpregnancy intervals on perinatal and neonatal outcomes in a multiethnic Pakistani population. Journal of tropical pediatrics. 2022;68(6):fmac088. doi: 10.1093/tropej/fmac088 36228309

[85] Regan AK, Ball SJ, Warren JL, Malacova E, Padula A, Marston C, et al. A Population-Based Matched-Sibling Analysis Estimating the Associations Between First Interpregnancy Interval and Birth Outcomes. Am J Epidemiol. 2019;188(1):9–16. doi: 10.1093/aje/kwy188 . 30188970 PMC6321799

[86] Nakamura Y, Tsuda H, Masahashi Y, Nakamura T, Suzuki M, Fukuhara N, et al. Impact of the interpregnancy interval after cesarean delivery on subsequent perinatal risks: a retrospective study. Archives of Gynecology and Obstetrics. 2023;308(2):479–85. doi: 10.1007/s00404-022-06651-9 35984489

[87] Tanigawa K, Ikehara S, Cui M, Kawanishi Y, Kimura T, Ueda K, et al. Association Between Interpregnancy Interval and Risk of Preterm Birth and Its Modification by Folate Intake: The Japan Environment and Children’s Study. Journal of Epidemiology. 2023;33(3):113–9. doi: 10.2188/jea.JE20210031 34024875 PMC9909173

[88] Reddy KM, Ravula SR, Palakollu S, Betha K. Prevalence of preterm birth and perinatal outcome: A rural tertiary teaching hospital-based study. Journal of Family Medicine and Primary Care. 2022;11(7):3909–14. doi: 10.4103/jfmpc.jfmpc_1440_21 36387651 PMC9648210

[89] Nisha MK, Alam A, Islam MT, Huda T, Raynes-Greenow C. Risk of adverse pregnancy outcomes associated with short and long birth intervals in Bangladesh: evidence from six Bangladesh Demographic and Health Surveys, 1996–2014. BMJ Open. 2019;9(2):e024392. doi: 10.1136/bmjopen-2018-024392 . 30798311 PMC6398728

[90] Ellison PT. Energetics and reproductive effort. American Journal of Human Biology. 2003;15(3):342–51. doi: 10.1002/ajhb.10152 12704710

[91] Smits LJ, Essed GG. Short interpregnancy intervals and unfavourable pregnancy outcome: role of folate depletion. The Lancet. 2001;358(9298):2074–7. doi: 10.1016/S0140-6736(01)07105-7 11755634

[92] Greenberg JA, Bell SJ, Guan Y, Yu Y-h. Folic acid supplementation and pregnancy: more than just neural tube defect prevention. Reviews in obstetrics and gynecology. 2011;4(2):52. 22102928 PMC3218540

[93] Witt WP, Wisk LE, Cheng ER, Hampton JM, Hagen EW. Preconception mental health predicts pregnancy complications and adverse birth outcomes: a national population-based study. Maternal and child health journal. 2012;16:1525–41. doi: 10.1007/s10995-011-0916-4 22124801 PMC3605892

[94] Molitoris J, Barclay K, Kolk M. When and where birth spacing matters for child survival: an international comparison using the DHS. Demography. 2019;56(4):1349–70. doi: 10.1007/s13524-019-00798-y 31270780 PMC6667399

[95] Getahun D, Strickland D, Ananth CV, Fassett MJ, Sacks DA, Kirby RS, et al. Recurrence of preterm premature rupture of membranes in relation to interval between pregnancies. American journal of obstetrics and gynecology. 2010;202(6):570. e1-. e6. doi: 10.1016/j.ajog.2009.12.010 20132922

[96] Conde-Agudelo A, Rosas-Bermúdez A, Kafury-Goeta AC. Effects of birth spacing on maternal health: a systematic review. American journal of obstetrics and gynecology. 2007;196(4):297–308. doi: 10.1016/j.ajog.2006.05.055 17403398

[97] Schummers L, Hutcheon JA, Hernandez-Diaz S, Williams PL, Hacker MR, VanderWeele TJ, et al. Association of short interpregnancy interval with pregnancy outcomes according to maternal age. JAMA internal medicine. 2018;178(12):1661–70. doi: 10.1001/jamainternmed.2018.4696 30383085 PMC6583597

[98] Wang Y, Zeng C, Chen Y, Yang L, Tian D, Liu X, et al. Short interpregnancy interval can lead to adverse pregnancy outcomes: A meta-analysis. Frontiers in Medicine. 2022;9:922053. doi: 10.3389/fmed.2022.922053 36530890 PMC9747778

[99] Coo H, Brownell MD, Ruth C, Flavin M, Au W, Day AG. Interpregnancy interval and adverse perinatal outcomes: a record-linkage study using the Manitoba population research data repository. Journal of Obstetrics and Gynaecology Canada. 2017;39(6):420–33. doi: 10.1016/j.jogc.2017.01.010 28363608

[100] King JC. The risk of maternal nutritional depletion and poor outcomes increases in early or closely spaced pregnancies. The Journal of nutrition. 2003;133(5):1732S–6S. doi: 10.1093/jn/133.5.1732S 12730491

[101] Goldenberg RL, Culhane JF, Johnson DC. Maternal infection and adverse fetal and neonatal outcomes. Clinics in perinatology. 2005;32(3):523–59. doi: 10.1016/j.clp.2005.04.006 16085019 PMC7119141

[102] Aaby P, Bukh J, Hoff G, Lisse IM, Smits AJ. Cross-sex transmission of infection and increased mortality due to measles. Reviews of infectious diseases. 1986;8(1):138–43. doi: 10.1093/clinids/8.1.138 3952421

[103] Garenne M, Aaby P. Pattern of exposure and measles mortality in Senegal. Journal of infectious diseases. 1990;161(6):1088–94. doi: 10.1093/infdis/161.6.1088 2345293

[104] Whitworth A, Stephenson R. Birth spacing, sibling rivalry and child mortality in India. Social science & medicine. 2002;55(12):2107–19. 12409124 10.1016/s0277-9536(02)00002-3

[105] Molitoris J. Feeling the Squeeze: The Effect of Birth Spacing on Infant and Child Mortality during the Demographic Transition. Available at SSRN 2773587. 2016.

[106] Pimentel J, Ansari U, Omer K, Gidado Y, Baba MC, Andersson N, et al. Factors associated with short birth interval in low-and middle-income countries: a systematic review. BMC pregnancy and childbirth. 2020;20(1):1–17. doi: 10.1186/s12884-020-2852-z 32164598 PMC7069040

[107] Chowdhury S, Singh A, Kasemi N, Chakrabarty M, Singh S. Short birth interval and associated factors in rural India: A cross-sectional study. Journal of Biosocial Science. 2023;55(4):735–54. doi: 10.1017/S0021932022000256 35787302

[108] Islam MZ, Islam MM, Rahman MM, Khan MN. Exploring hot spots of short birth intervals and associated factors using a nationally representative survey in Bangladesh. Scientific Reports. 2022;12(1):9551. doi: 10.1038/s41598-022-13193-2 35680970 PMC9184619

[109] Hutcheon JA, Moskosky S, Ananth CV, Basso O, Briss PA, Ferré CD, et al. Good practices for the design, analysis, and interpretation of observational studies on birth spacing and perinatal health outcomes. Paediatric and Perinatal Epidemiology. 2019;33(1):O15–O24. doi: 10.1111/ppe.12512 30311958 PMC6378590

