# Supplementary material for: Effects of short inter-pregnancy/birth interval on adverse perinatal outcomes in Asia-Pacific region: A systematic review and meta-analysis
Source: PLoS One. 2024 Jul 31;19(7):e0307942. doi: 10.1371/journal.pone.0307942 (PMC11290688; doi:10.1371/journal.pone.0307942)
Supplement: S1 File — (DOCX) [file pone.0307942.s001.docx]

**Supplementary material for the effects of short inter-pregnancy/birth interval on adverse perinatal outcomes in Asia-Pacific Region: a systematic review and meta-analysis.**

**Table S1. Search strategy of Medline database (September 2000 to May 2023)**

| **#** | **Query** | **Results** |
| --- | --- | --- |
| 1 | wom?[n.mp](http://n.mp/). or Wom?n/ | 1,380,889 |
| 2 | women of reproductive [age.mp](http://age.mp/). | 10,272 |
| 3 | [mother.mp](http://mother.mp/). or Mothers/ | 176,472 |
| 4 | Infant, Newborn/ or neonat*.mp. | 817,400 |
| 5 | Infant, Newborn/ or newborn*.mp. | 833,557 |
| 6 | children aged less than 28 [days.mp](http://days.mp/). | 0 |
| 7 | Infant/ or infant*.mp. | 1,391,006 |
| 8 | less than one year child*.mp. | 3 |
| 9 | children aged less than 12 [months.mp](http://months.mp/). | 18 |
| 10 | children less than 12 months*.mp. | 75 |
| 11 | under-five child*.mp. | 1,688 |
| 12 | under-5 [child.mp](http://child.mp/). | 89 |
| 13 | less than five years [child.mp](http://child.mp/). | 0 |
| 14 | Child/ or child*.mp. or Child Health/ | 2,745,473 |
| 15 | children aged less than 59 month*.mp. | 2 |
| 16 | children less than 59 month*.mp. | 3 |
| 17 | 1 or 2 or 3 or 4 or 5 or 6 or 7 or 8 or 9 or 10 or 11 or 12 or 13 or 14 or 15 or 16 | 4,729,513 |
| 18 | "Birth Intervals"/ or "birth interval*".mp. | 2,991 |
| 19 | birth spacing*.mp. | 1,135 |
| 20 | pregnancy interval*.mp. | 459 |
| 21 | interpregnancy interval*.mp. | 614 |
| 22 | inter-pregnancy interval*.mp. | 179 |
| 23 | interbirth interval*.mp. | 301 |
| 24 | inter-birth interval*.mp. | 123 |
| 25 | rapid repeat [pregnancy.mp](http://pregnancy.mp/). | 68 |
| 26 | pregnancy spacing*.mp. | 92 |
| 27 | repeat teenage [pregnancy.mp](http://pregnancy.mp/). | 10 |
| 28 | child spacing*.mp. | 323 |
| 29 | birth to birth interval*.mp. | 49 |
| 30 | birth to pregnancy interval*.mp. | 13 |
| 31 | pregnancy to pregnancy interval*.mp. | 7 |
| 32 | 18 or 19 or 20 or 21 or 22 or 23 or 24 or 25 or 26 or 27 or 28 or 29 or 30 or 31 | 4,610 |
| 33 | American Samoa.mp. or American Samoa/ | 456 |
| 34 | Afghanistan.mp. or Afghanistan/ | 8,179 |
| 35 | Bangladesh.mp. or Bangladesh/ | 21,323 |
| 36 | Bhutan.mp. or Bhutan/ | 1,255 |
| 37 | Cambodia.mp. or Cambodia/ | 5,762 |
| 38 | China.mp. or China/ | 380,043 |
| 39 | Fiji.mp. or Fiji/ | 2,561 |
| 40 | India.mp. or India/ | 181,305 |
| 41 | Indonesia.mp. or Indonesia/ | 22,358 |
| 42 | Kiribati.mp. or Micronesia/ | 1,423 |
| 43 | Democratic People's Republic of Korea.mp. or "Democratic People's Republic of Korea"/ | 379 |
| 44 | Lao People's Democratic Republic.mp. | 665 |
| 45 | Malaysia.mp. or Malaysia/ | 27,554 |
| 46 | Maldives.mp. or Indian Ocean Islands/ | 1,173 |
| 47 | Marshall Islands.mp. or Micronesia/ | 1,438 |
| 48 | the Federated States of Micronesia.mp. | 287 |
| 49 | Mongolia.mp. or Mongolia/ | 6,202 |
| 50 | Myanmar.mp. or Myanmar/ | 5,562 |
| 51 | Nepal.mp. or Nepal/ | 15,491 |
| 52 | Pakistan.mp. or Pakistan/ | 33,825 |
| 53 | Papua New Guinea.mp. or Papua New Guinea/ | 5,840 |
| 54 | Philippines.mp. or Philippines/ | 14,476 |
| 55 | Samoa/ or Samoa.mp. | 1,347 |
| 56 | Sri Lanka.mp. or Sri Lanka/ | 10,336 |
| 57 | Solomon Islands.mp. or Melanesia/ | 1,590 |
| 58 | Thailand.mp. or Thailand/ | 43,721 |
| 59 | Timor-Leste.mp. or Timor-Leste/ | 544 |
| 60 | Tonga/ or Tonga.mp. | 628 |
| 61 | Tuvalu.mp. or Micronesia/ | 1,332 |
| 62 | Vanuatu.mp. or Vanuatu/ | 849 |
| 63 | Vietnam.mp. or Vietnam/ | 23,326 |
| 64 | Asia-Pacific region*.mp. | 2,514 |
| 65 | Asia-Pacific [countries.mp](http://countries.mp/). | 348 |
| 66 | Asia-Pacific [state.mp](http://state.mp/). | 0 |
| 67 | Australia.mp. or Australia/ | 194,141 |
| 68 | Brunei Darussalam.mp. | 280 |
| 69 | French Polynesia.mp. or Polynesia/ | 2,537 |
| 70 | Guam.mp. or Guam/ | 1,486 |
| 71 | Hong Kong.mp. or Hong Kong/ | 28,137 |
| 72 | Japan.mp. or Japan/ | 225,242 |
| 73 | Republic of Korea.mp. or "Republic of Korea"/ | 44,002 |
| 74 | Macao SAR.mp. | 37 |
| 75 | Nauru.mp. or Micronesia/ | 1,369 |
| 76 | New Caledonia.mp. or New Caledonia/ | 1,851 |
| 77 | New Zealand.mp. or New Zealand/ | 81,754 |
| 78 | Northern Mariana Islands.mp. or Micronesia/ | 1,363 |
| 79 | Palau.mp. or Palau/ | 510 |
| 80 | Singapore.mp. or Singapore/ | 23,549 |
| 81 | Singapore.mp. or Singapore/ | 23,549 |
| 82 | Singapore.mp. or Singapore/ | 23,549 |
| 83 | 33 or 34 or 35 or 36 or 37 or 38 or 39 or 40 or 41 or 42 or 43 or 44 or 45 or 46 or 47 or 48 or 49 or 50 or 51 or 52 or 53 or 54 or 55 or 56 or 57 or 58 or 59 or 60 or 61 or 62 or 63 or 64 or 65 or 66 or 67 or 68 or 69 or 70 or 71 or 72 or 73 or 74 or 75 or 76 or 77 or 78 or 79 or 80 or 81 or 82 | 1,309,908 |
| 84 | 17 and 32 and 83 | 1,010 |
| 85 | limit 84 to (english language and yr="2000 -Current") | 470 |

**Table S2. Search strategy of Web of Science database (September 2000 to May 2023)**

| **#** | **Search** | **Result** |
| --- | --- | --- |
| 1 | ALL=(wom?n OR "women of reproductive age" OR mother* OR neonat* OR newborn* OR "children aged less than 28 days" OR infant* OR "less than one year child*" OR "children aged less than 12 months" OR "children less than 12 months*" OR "under-five child*" OR "under-5 child" OR "less than five years child" OR child* OR "children aged less than 59 month*" OR "children less than 59 month*" ) | 5,428,406 |
| 2 | ALL=("birth interval*" OR "birth spacing*" OR "pregnancy interval*" OR "interpregnancy interval*" OR "inter-pregnancy interval*" OR "interbirth interval*" OR "inter-birth interval*" OR "rapid repeat pregnancy" OR "pregnancy spacing*" OR "repeat teenage pregnancy" OR "child spacing*" OR "birth to birth interval*" OR "birth to pregnancy interval*" OR "pregnancy to pregnancy interval*") | 3,794 |
| 3 | ALL=("american samoa" OR afghanistan OR Australia OR bangladesh OR bhutan OR "Brunei Darussalam" OR cambodia OR china OR fiji OR "French Polynesia" OR Guam OR "Hong Kong" OR india OR indonesia OR Japan OR kiribati OR "Republic of Korea" OR "democratic people's republic of korea" OR "lao people's democratic republic" OR "Macao SAR" OR malaysia OR maldives OR "marshall islands" OR "the federated states of micronesia" OR mongolia OR myanmar OR Nauru OR nepal OR "New Caledonia" OR "New Zealand" OR "Northern Mariana Islands" OR pakistan OR "papua new guinea" OR palau OR philippines OR samoa OR singapore OR "sri lanka" OR "solomon islands" OR thailand OR "timor-leste" OR tonga OR tuvalu OR vanuatu OR vietnam OR "asia-pacific region*" OR "asia-pacific countries" OR "asia-pacific state" ) | 19,203,217 |
| 1&2&3 | (Filtered by year (01-09-2000 to 30-05-2023) and English language | 919 |
| Total |  |  |

**Table S3. Search strategy of CINAHL database (September 2000 to May 2023)**

| **#** | **Search** | **Result** |
| --- | --- | --- |
| 1 | (wom?n OR "women of reproductive age" OR mother* OR neonat* OR newborn* OR "children aged less than 28 days" OR infant* OR "less than one year child*" OR "children aged less than 12 months" OR "children less than 12 months*" OR "under-five child*" OR "under-5 child" OR "less than five years child" OR child* OR "children aged less than 59 month*" OR "children less than 59 month*" ) | 1,493,787 |
| 2 | ("birth interval*" OR "birth spacing*" OR "pregnancy interval*" OR "interpregnancy interval*" OR "inter-pregnancy interval*" OR "interbirth interval*" OR "inter-birth interval*" OR "rapid repeat pregnancy" OR "pregnancy spacing*" OR "repeat teenage pregnancy" OR "child spacing*" OR "birth to birth interval*" OR "birth to pregnancy interval*" OR "pregnancy to pregnancy interval*") | 3,525 |
| 3 | ("american samoa" OR afghanistan OR Australia OR bangladesh OR bhutan OR "Brunei Darussalam" OR cambodia OR china OR fiji OR "French Polynesia" OR Guam OR "Hong Kong" OR india OR indonesia OR Japan OR kiribati OR "Republic of Korea" OR "democratic people's republic of korea" OR "lao people's democratic republic" OR "Macao SAR" OR malaysia OR maldives OR "marshall islands" OR "the federated states of micronesia" OR mongolia OR myanmar OR Nauru OR nepal OR "New Caledonia" OR "New Zealand" OR "Northern Mariana Islands" OR pakistan OR "papua new guinea" OR palau OR philippines OR samoa OR singapore OR "sri lanka" OR "solomon islands" OR thailand OR "timor-leste" OR tonga OR tuvalu OR vanuatu OR vietnam OR "asia-pacific region*" OR "asia-pacific countries" OR "asia-pacific state" ) | 421,677 |
| 1&2&3 | Filtered by year of publication (2000-2023) AND Filtered by language (English) | 457 |
| Total |  |  |

**Table S4. Search strategy of Scopus database (September 2000 to May 2023)**

| **#** | **Search** | **Result** |
| --- | --- | --- |
| 1 | (wom?n OR "women of reproductive age" OR mother* OR neonat* OR newborn* OR "children aged less than 28 days" OR infant* OR "less than one year child*" OR "children aged less than 12 months" OR "children less than 12 months*" OR "under-five child*" OR "under-5 child" OR "less than five years child" OR child* OR "children aged less than 59 month*" OR "children less than 59 month*" ) | 6,468,365 |
| 2 | ("birth interval*" OR "birth spacing*" OR "pregnancy interval*" OR "interpregnancy interval*" OR "inter-pregnancy interval*" OR "interbirth interval*" OR "inter-birth interval*" OR "rapid repeat pregnancy" OR "pregnancy spacing*" OR "repeat teenage pregnancy" OR "child spacing*" OR "birth to birth interval*" OR "birth to pregnancy interval*" OR "pregnancy to pregnancy interval*") | 5694 |
| 3 | ("american samoa" OR afghanistan OR Australia OR bangladesh OR bhutan OR "Brunei Darussalam" OR cambodia OR china OR fiji OR "French Polynesia" OR Guam OR "Hong Kong" OR india OR indonesia OR Japan OR kiribati OR "Republic of Korea" OR "democratic people's republic of korea" OR "lao people's democratic republic" OR "Macao SAR" OR malaysia OR maldives OR "marshall islands" OR "the federated states of micronesia" OR mongolia OR myanmar OR Nauru OR nepal OR "New Caledonia" OR "New Zealand" OR "Northern Mariana Islands" OR pakistan OR "papua new guinea" OR palau OR philippines OR samoa OR singapore OR "sri lanka" OR "solomon islands" OR thailand OR "timor-leste" OR tonga OR tuvalu OR vanuatu OR vietnam OR "asia-pacific region*" OR "asia-pacific countries" OR "asia-pacific state" ) | 4,087,211 |
| 1&2&3 | Filtered by year of publication (2000-2023) AND Filtered by language (English) | 655 |
| Total |  |  |

**Table S5. Search strategy of Maternity and Infant Care database (September 2000 to May 2023)**

| **#** | **Searches** | **Results** |
| --- | --- | --- |
| 1 | wom?n.mp. [mp=abstract, heading word, title] | 121203 |
| 2 | "women of reproductive age".mp. [mp=abstract, heading word, title] | 1011 |
| 3 | mother*.mp. [mp=abstract, heading word, title] | 56170 |
| 4 | neonat*.mp. [mp=abstract, heading word, title] | 56595 |
| 5 | newborn*.mp. [mp=abstract, heading word, title] | 44440 |
| 6 | "children aged less than 28 days".mp. [mp=abstract, heading word, title] | 0 |
| 7 | infant*.mp. [mp=abstract, heading word, title] | 99941 |
| 8 | "less than one year child*".mp. [mp=abstract, heading word, title] | 0 |
| 9 | "children aged less than 12 months".mp. [mp=abstract, heading word, title] | 1 |
| 10 | "children less than 12 month*".mp. [mp=abstract, heading word, title] | 5 |
| 11 | "under-five child*".mp. [mp=abstract, heading word, title] | 74 |
| 12 | "under-5 child".mp. [mp=abstract, heading word, title] | 13 |
| 13 | "less than five years child*".mp. [mp=abstract, heading word, title] | 0 |
| 14 | child*.mp. [mp=abstract, heading word, title] | 72123 |
| 15 | "children aged less than 59 month*".mp. [mp=abstract, heading word, title] | 0 |
| 16 | "children less than 59 month*".mp. [mp=abstract, heading word, title] | 0 |
| 17 | 1 or 2 or 3 or 4 or 5 or 6 or 7 or 8 or 9 or 10 or 11 or 12 or 13 or 14 or 15 or 16 | 236863 |
| 18 | "birth interval*".mp. [mp=abstract, heading word, title] | 420 |
| 19 | "birth spac*".mp. [mp=abstract, heading word, title] | 194 |
| 20 | "pregnancy interv*".mp. [mp=abstract, heading word, title] | 287 |
| 21 | "interpregnancy interv*".mp. [mp=abstract, heading word, title] | 328 |
| 22 | "inter-pregnancy interv*".mp. [mp=abstract, heading word, title] | 83 |
| 23 | "interbirth interv*".mp. [mp=abstract, heading word, title] | 20 |
| 24 | "inter-birth interv*".mp. [mp=abstract, heading word, title] | 17 |
| 25 | "rapid repeat pregnan*".mp. [mp=abstract, heading word, title] | 37 |
| 26 | "pregnancy spac*".mp. [mp=abstract, heading word, title] | 33 |
| 27 | "repeat teenage pregnan*".mp. [mp=abstract, heading word, title] | 7 |
| 28 | "child spac*".mp. [mp=abstract, heading word, title] | 57 |
| 29 | "birth to birth interv*".mp. [mp=abstract, heading word, title] | 11 |
| 30 | "birth to pregnancy interv*".mp. [mp=abstract, heading word, title] | 6 |
| 31 | "pregnancy to pregnancy interv*".mp. [mp=abstract, heading word, title] | 3 |
| 32 | 18 or 19 or 20 or 21 or 22 or 23 or 24 or 25 or 26 or 27 or 28 or 29 or 30 or 31 | 1146 |
|  | American Samoa.mp. [mp=abstract, heading word, title] | 6 |
|  | Afghanistan.mp. [mp=abstract, heading word, title] | 226 |
|  | Bangladesh.mp. [mp=abstract, heading word, title] | 999 |
|  | Bhutan.mp. [mp=abstract, heading word, title] | 33 |
|  | Cambodia.mp. [mp=abstract, heading word, title] | 161 |
|  | China.mp. [mp=abstract, heading word, title] | 2658 |
|  | Fiji.mp. [mp=abstract, heading word, title] | 38 |
|  | India.mp. [mp=abstract, heading word, title] | 2565 |
|  | Indonesia.mp. [mp=abstract, heading word, title] | 514 |
|  | Kiribati.mp. [mp=abstract, heading word, title] | 8 |
|  | Democratic People's Republic of Korea.mp. [mp=abstract, heading word, title] | 1 |
|  | Lao People's Democratic Republic.mp. [mp=abstract, heading word, title] | 37 |
|  | "Marshall Islands".mp. [mp=abstract, heading word, title] | 7 |
|  | "the Federated States of Micronesia".mp. [mp=abstract, heading word, title] | 2 |
|  | "Mongolia".mp. [mp=abstract, heading word, title] | 47 |
|  | Myanmar.mp. [mp=abstract, heading word, title] | 126 |
|  | Nepal.mp. [mp=abstract, heading word, title] | 719 |
|  | Pakistan.mp. [mp=abstract, heading word, title] | 779 |
|  | "Papua New Guinea".mp. [mp=abstract, heading word, title] | 139 |
|  | Philippines.mp. [mp=abstract, heading word, title] | 323 |
|  | Samoa.mp. [mp=abstract, heading word, title] | 25 |
|  | "Sri Lanka".mp. [mp=abstract, heading word, title] | 213 |
|  | "Solomon Islands".mp. [mp=abstract, heading word, title] | 16 |
|  | Thailand.mp. [mp=abstract, heading word, title] | 566 |
|  | "Timor-Leste".mp. [mp=abstract, heading word, title] | 29 |
|  | Tonga.mp. [mp=abstract, heading word, title] | 8 |
|  | Tuvalu.mp. [mp=abstract, heading word, title] | 3 |
|  | Vietnam.mp. [mp=abstract, heading word, title] | 356 |
|  | "Asia-Pacific region*".mp. [mp=abstract, heading word, title] | 36 |
|  | "Asia-Pacific countr*".mp. [mp=abstract, heading word, title] | 3 |
|  | "Asia-Pacific stat*".mp. [mp=abstract, heading word, title] | 0 |
|  | 33 or 34 or 35 or 36 or 37 or 38 or 39 or 40 or 41 or 42 or 43 or 44 or 45 or 46 or 47 or 48 or 49 or 50 or 51 or 52 or 53 or 54 or 55 or 56 or 57 or 58 or 59 or 60 or 61 or 62 or 63 | 9417 |
|  | 17 and 32 and 64 | 100 |
|  | limit 65 to yr="2000 -Current" | 113 |

**Table S6: Summary of studies included to investigate the effects of short inter-pregnancy/birth interval on adverse perinatal outcomes in the Asia-Pacific Region**

| **Author, year** | **Country** | **Design** | **Population characteristics** | **Measured adverse perinatal outcomes** | **Classification (in months), interval type, and terminology used** | **Key findings** |
| --- | --- | --- | --- | --- | --- | --- |
| Arshad et al 2021[1] | Pakistan | Prospective cohort | 420 women with singleton pregnancies and gestational age of >28 weeks | LBW and PTB | <6: birth to pregnancy; interpregnancy interval (IPI) | A short interpregnancy interval was associated with LBW and PTB (P<0.001) |
| Ball et al 2014[2] | Australia | Retrospective cohort | 40, 441 mothers who had their first three births as a liveborn singleton | LBW, PTB, and SGA | <6: birth to pregnancy; IPI | A short interpregnancy interval of less than six months showed a weaker effect on LBW (AOR=1.03;95%CI: 0.79,1.34), PTB (AOR=1.07;95%CI: 0.86,1.34), SGA (AOR=1.08; 95%CI: 0.87,1.34) |
| Borah et al 2016[3] | India | Cross-sectional | 450 singleton infants | LBW | <18: not defined; IPI | The odds of being LBW were found to be approximately four times (OR=3.93; 95 % CI: 2.16, 7.13) higher among babies born within short interpregnancy interval |
| Chowdhury et al2018[4] | Bangladesh | Cross-sectional | 8,588 children born singleton | Small birth size | <33: birth to birth; birth interval (BI) | Birth interval was significantly associated with small birth size (OR=1.01; 95 % CI: 0.98, 1.05) |
| DasGupta et al 2019[5] | Afghanistan | Cross-sectional | 2, 773 children born in healthy facilities | LBW | <24: Pregnancy to pregnancy; BI | Short birth interval was not significantly associated with an increased risk of low birth weight (OR=0.80; 95 % CI: 0.40, 1.79) |
| deJonge et al 2014[6] | Bangladesh | Cross-sectional | 5,571 women with complete information on birth interval, pregnancy outcomes, and predictors | Adverse outcome of pregnancy,  perinatal mortality,  neonatal mortality,  stillbirths, and early neonatal mortality | <33^*^: birth to birth; BI | Very short birth intervals of less than 21 months were associated with an increased risk of adverse outcomes of pregnancy (AOR=2.33; 95% CI: 1.51, 3.29), perinatal mortality (AOR=2.33; 95% CI: 1.55, 3.50), neonatal mortality (AOR=2.28; 95% CI: 1.28, 4.05), stillbirth rate (AOR=2.13; 95% CI: 1.28, 3.53),, and were not significantly associated with an increased risk of early neonatal mortality  (AOR=2.59; 95% CI: 0.53, 2.73). |
| Fatima et al 2021[7] | Pakistan | Cross-sectional | 190 women with singleton uncomplicated pregnancies | PTB | <18: not defined; IPI | The rate of preterm was significantly higher in those women who had short inter-interpregnancy intervals (p=0.0005) |
| Hanif et al 2019[8] | Pakistan | Case-control | 953 pregnant women with parity greater than one | PTB | <12: not defined; IPI | An interpregnancy interval of < 12 months was significantly associated with an increased risk of PTB (AOR=1.5; 95% CI: 1.13, 2.00) |
| Hosain et al 2006[9] | Bangladesh | Cohort | 350 pregnant women | LBW | <24: not defined; BI | Short birth interval was not statistically associated with an increased risk of LBW (AOR=1.32; 95% CI: 0.57, 3.03) |
| Huo et al 2013[10] | China | Prospective cohort | 4, 682 nulliparous women with one mifepristone-induced abortion in their first pregnancy | LBW, PTB, and SGA | <24: not defined; IPI | An interpregnancy interval of < 6 months was not statistically associated with an increased risk of LBW (AOR=1.43; 95%CI: 0.64, 3.17), PTB (AOR=0.64; 95%CI: 0.34, 1.19). The odds of being SGA were two times higher (AOR=2.01; 95%CI: 1.04, 3.88) among babies born within an interpregnancy interval of <6 months. |
| Hussain et al 2002[11] | Pakistan | Cross-sectional | 4, 488 singleton live births born to 912 ever-married women | Neonatal mortality | <6: not defined; BI | Preceding interval of < 6 months was associated with an increased risk of neonatal mortality (AOR=1.80; 95%CI: 1.10, 3.0) |
| Ismah et al2018[12] | Indonesia | Cross-sectional | 752 pregnant women with a gestational age of ≥24 weeks | Low fetal weight | <23: not defined; IPI | Short interpregnancy interval associated with an increased incident of low fetal weight (PR=4.85; 95%: 1.92, 12.24) |
| Kader et al 2014[13] | India | Cross-sectional | 20, 946 infants born to women who gave birth at least once during 5 years preceding the survey | LBW | <18: not defined; IPI | Short interpregnancy interval (<18 months) was not statistically associated with an elevated risk of LBW (AOR=1.08, 95%CI: 0.95, 1.24) |
| Kannaujiya et al 2020[14] | India | Cross-sectional | 52,825 most recent births | LBW | <6: birth to pregnancy; IPI | The risk of LBW was significantly higher among births whose mothers had interpregnancy interval < 6 months (AOR=1.19; 95% CI:1.05, 1.36) |
| Kaur et al 2014[15] | India | Cross-sectional | 271 mothers who were admitted to the hospital with a gestational age of >28 weeks and delivered singleton baby | LBW | <18: pregnancy to pregnancy; IPI | A short interpregnancy interval of <18 months was associated with an increased risk of LBW (p<0.01) |
| Kibria et al 2018[16] | Afghanistan | Cross-sectional | 19, 636 children/neonates | Early neonatal mortality | <24: pregnancy to pregnancy; BI | Short birth interval was significantly associated with an increased risk of early neonatal (AOR=2.6; 95% CI: 1.4, 4.9). |
| Kumar et al 2005[17] | India | Cross-sectional | 1,350 pregnant women | LBW | <24: pregnancy to pregnancy; IPI | A short interpregnancy interval of < two years was associated with an increased risk of LBW |
| Memon et al 2005[18] | Pakistan | Case-control | 160 newborns | LBW | <12: not defined, BI | The odds of being LBW were higher among babies born within a short birth interval of <12 months (p<0.01) |
| Metgud et al 2012[19] | India | Cross-sectional | 1,138 pregnant women | LBW | <24: birth to pregnancy; BI | The odds of being low birth weight were approximately two times (AOR=2.4; 95%CI: 1.30, 4.60) higher among newborn babies born within short birth interval. |
| Mohsin et al 2008[20] | Australia | Cross-sectional | 244, 840 mothers with two singleton births | LBW and PTB | <12: birth to birth; BI | Short birth interval was associated with an increased risk of LBW (AOR=1.65; 95%CI: 1.43, 1.91) and PTB (AOR=3.60; 95%CI: 3.27, 3.97) |
| Nagargoje et al 2011[21] | India | Case-control | 860 women who came for delivery | LBW | <24: not defined; BI | The odds of being LBW were approximately two times higher (OR=1.81; 95%CI:1.10, 2.98) among babies born within short birth interval |
| Negi et al 2006[22] | India | Prospective cohort | 172 pregnant women | LBW | <12: not defined; IPI | Short interpregnancy interval was associated with an elevated risk of LBW (OR=2.58) |
| Patel et al 2021[23] | India | Cross-sectional | 3,146** | Neonatal mortality | <24: not defined; BI | Short birth interval was associated with a reduced risk of neonatal mortality (aHR=0.61; 95%CI: 0.54, 0.67) |
| Peters et al 2001[24] | Papua New Guinea | Case-control | 299 neonates | LBW | <24: birth to birth; BI | Short birth interval was associated with an increased risk of being LBW. |
| Regan et al 2019[25] | Australia | Retrospective cohort | 174, 200 first and second births | LBW, PTB, and SGA | <6: birth to pregnancy; IPI | Interpregnancy intervals of <6 months were not significantly associated with LBW (adjusted interaction odds ratio=1.00; 95%CI: 0.81, 1.29) and SGA (adjusted interaction odds ratio=1.01, 95%CI: 0.86, 1.16). The odds of PTB were higher for siblings born following an interpregnancy interval of <6 months (adjusted interaction odds ratio=1.22, 95%CI: 1.06, 1.38) |
| Shi et al 2021[26] | China | Cross-sectional | 13, 231 non-primi women with live births | LBW, PTB, SGA, and birth defects | <6: birth to pregnancy; IPI | A short interpregnancy interval of <6 months was statistically associated with SGA (RR=1.25; 95% CI: 1.04, 1.52) and birth defects (RR=2.55; 95% CI: 1.45, 4.47, but not with LBW (RR=1.37; 95% CI: 0.88, 2.13) and PTB (RR=1.10; 95% CI: 0.66, 1.82). |
| Tanigawa et al 2021[27] | Japan | Retrospective cohort | 55,203 singleton live birth pregnancies | PTB | <6: birth to pregnancy; IPI | A short interpregnancy interval of <6 months was associated with PTB (AOR=1.63; 95%CI: 1.30, 2.04) |
| Williams et al 2008[28] | India | Cross-sectional | 80, 164 births | Stillbirth and neonatal death | <18: not defined; BI | The odds of experiencing stillbirth and neonatal death were about three times (AOR=3.10; 95%CI: 2.69, 3.57) and four (AOR=4.12; 95%CI: 3.74, 4.55) times higher, respectively, among births recorded to women with short birth intervals. |
| Xu et al 2022[29] | China | Cross-sectional | 725, 392 first and second-born sibling pairs of multiparous mothers | LBW. PTB, and SGA | <6: birth to pregnancy; IPI | A short interpregnancy interval of 6 months was associated with higher risks of LBW (AOR=1.88; 95% CI: 1.79, 1.98), PTB (AOR =1.96; 95% CI: 1.87, 2.06), and SGA (AOR=1.34; 95% CI: 1.30, 1.38) |
| Yamashita et al 2015[30] | Japan | Retrospective cohort | 547 women with previous PTB | Preterm birth/spontaneous | <12: birth to pregnancy; IPI | A short interpregnancy interval of <12 months was associated with an increased risk of PTB (AOR =2.13; 95%CI: 1.17, 3.85) |
| Zhang et al 2018[31] | China | Retrospective cohort | 227,352 women with their singleton first and second delivery | PTB | <6: birth to pregnancy; IPI | A short interpregnancy interval of <6 months was associated with higher risks of preterm (aRR=2.04; 95%CI: 1.83,2.27) |
| Zhang et al 2012[32] | China | Case-control | 2,782 women with preterm and term delivery (1:1) | PTB | ≤6: birth to pregnancy; IPI | Interpregnancy interval was not associated with preterm birth. |
| Agrawal et al2016[33] | India | Cross-sectional | 13, 612 women with singleton live births | Neonatal mortality | <18: not defined; BI | The likelihood of neonatal mortality was  almost twice (OR=1.87; 95% CI:1.33, 2.61) among the newborn whose birth  the interval was less than 18 months |
| Asif et al 2022[34] | Pakistan | Cross-sectional | 2,246 women who reported their child health variable information in the PDHS | Child health/child weight at birth | <33: birth to birth; BI (spacing) | Birth spacing was found to be a strong predictor for improving a child’s health |
| Latif et al 2019[35] | Pakistan | Cross-sectional | 300 women | LBW, PTB, intrauterine growth restriction, and  nursery admission | <18: Pregnancy to pregnancy: IPI | A short interpregnancy interval of <18 months was not significantly associated with LBW, PTB, IUGR, and nursery admission |
| Nakamura et al 2022[36] | Japan | Retrospective cohort | 592 births occurred after caesarean delivery | PTB and fetal growth restriction | <18: birth to pregnancy; IPI | No significant differences were observed in the risks of PTB and fetal growth restriction among all interpregnancy interval groups. |
| Acharya et al 2022[37] | Nepal | Cross-sectional | 305 mothers who had children under the age of 6 months | PTB | <24: not defined; BI | A short birth interval was associated with an increased risk of PTB (AOR=5.16; 95% CI: 1.62, 16.4) |
| Tanigawa et al 2023[38] | Japan | Retrospective cohort | 55,203 singleton live-birth pregnancies | PTB | <6: birth to pregnancy; IPI | A short interpregnancy interval of <6 months was statistically associated with lPTB (AOR=1.76; 95% CI: 1.35, 2.29) |
| Reddy et al 2022[39] | India | Case-control | 383 preterm and term neonates (case vs control) | PTB | ≤12: not defined; IPI | A short interpregnancy interval of <12 months was associated with an increased risk of PTB (AOR = 2.78; 95%CI: 1.13, 6.84) |
| Murtaza et al 2022[40] | Pakistan | Cross-sectional | 2,798 women who were admitted for delivery and gave birth to a singleton pregnancy | Neonatal death and perinatal death | <24: birth to birth; BI | Women with short birth interval had increased odds of neonatal death [adjusted odd ratio (AOR=1.47) and perinatal death [adjusted odd ratio (AOR=1.50) |
| Bera et al 2023[41] | India | Prospective cohort | 173 multigravida women carrying a singleton pregnancy having reasonable information and records of previous and current pregnancy and having at least three antenatal visits during the present pregnancy were included in the present study | PTB | <24: not defined; BI | A short birth interval was associated with a higher incidence of PTB (p=0.041) |

AOR: adjusted odds ratio; BI: birth interval; IPI: inter-pregnancy interval; LBW: low birthweight; PTB: preterm birth; RR: relative risk; SGA: small for gestational age

**Risk of bias assessment of the included studies**

**Table S7:** Joanna Briggs Institute risk of bias assessment for **Cross-sectional studies**

| S.No | Authors/year | Items | | | | | | | | | | | | | | | | | | | | | | | | Total | Judgment |
| --- | --- | --- | --- | --- | --- | --- | --- | --- | --- | --- | --- | --- | --- | --- | --- | --- | --- | --- | --- | --- | --- | --- | --- | --- | --- | --- | --- |
|  |  | Item 1 | | | Item 2 | | | Item 3 | | | Item 4 | | | Item 5 | | | Item 6 | | | Item 7 | | | Item 8 | | |  |  |
|  | Borah et al 2016 | Y |  |  | Y |  |  | Y |  |  | Y |  |  | Y |  |  | Y |  |  | Y |  |  | Y |  |  | 8 | Low |
|  | Chowdhury et al2018 | Y |  |  | Y |  |  | Y |  |  | Y |  |  | Y |  |  | Y |  |  | Y |  |  | Y |  |  | 8 | Low |
|  | DasGupta et al 2019 | N |  |  | Y |  |  | N |  |  | Y |  |  | Y |  |  | Y |  |  | Y |  |  | Y |  |  | 6 | Medium |
|  | DeJonge et al 2014 | Y |  |  | Y |  |  | Y |  |  | N |  |  | Y |  |  | N |  |  | Y |  |  | Y |  |  | 6 | Medium |
|  | Fatima et al 2021 | Y |  |  | Y |  |  | N |  |  | N |  |  | N |  |  | N |  |  | Y |  |  | N |  |  | 3 | High |
|  | Hussain et al 2002 | Y |  |  | Y |  |  | Y |  |  | Y |  |  | Y |  |  | Y |  |  | Y |  |  | Y |  |  | 8 | Low |
|  | Ismah et al2018 | Y |  |  | N |  |  | N |  |  | Y |  |  | N |  |  | N |  |  | Y |  |  | U |  |  | 3 | High |
|  | Kader et al 2014 | Y |  |  | Y |  |  | N |  |  | Y |  |  | Y |  |  | Y |  |  | Y |  |  | Y |  |  | 7 | Low |
|  | Kannaujiya et al 2020 | Y |  |  | Y |  |  | Y |  |  | Y |  |  | Y |  |  | Y |  |  | Y |  |  | Y |  |  | 8 | Low |
|  | Kaur et al 2014 | Y |  |  | Y |  |  | Y |  |  | N |  |  | Y |  |  | Y |  |  | Y |  |  | Y |  |  | 7 | Low |
|  | Kibria et al 2018 | Y |  |  | Y |  |  | Y |  |  | Y |  |  | Y |  |  | Y |  |  | Y |  |  | Y |  |  | 8 | Low |
|  | Kumar et al 2005 | N |  |  | Y |  |  | N |  |  | N |  |  | Y |  |  | N |  |  | N |  |  | N |  |  | 2 | High |
|  | Metgud et al 2012 | Y |  |  | Y |  |  | N |  |  | Y |  |  | N |  |  | N |  |  | Y |  |  | Y |  |  | 5 | Medium |
|  | Mohsin et al 2008 | Y |  |  | Y |  |  | Y |  |  | N |  |  | Y |  |  | Y |  |  | Y |  |  | Y |  |  | 8 | Low |
|  | Patel et al 2021 | N |  |  | N |  |  | Y |  |  | N |  |  | Y |  |  | Y |  |  | N |  |  | Y |  |  | 4 | High |
|  | Shi et al 2021 | Y |  |  | Y |  |  | Y |  |  | Y |  |  | Y |  |  | Y |  |  | Y |  |  | Y |  |  | 8 | Low |
|  | Williams et al 2008 | Y |  |  | Y |  |  | Y |  |  | Y |  |  | Y |  |  | Y |  |  | Y |  |  | Y |  |  | 8 | Low |
|  | Xu et al 2022 | Y |  |  | Y |  |  | Y |  |  | Y |  |  | Y |  |  | Y |  |  | Y |  |  | Y |  |  | 8 | Low |
|  | Agrawal et al2016 | Y |  |  | Y |  |  | N |  |  | N |  |  | Y |  |  | Y |  |  | Y |  |  | Y |  |  | 6 | Medium |
|  | Asif et al 2022 | Y |  |  | Y |  |  | Y |  |  | N |  |  | Y |  |  | Y |  |  | N |  |  | Y |  |  | 6 | Medium |
|  | Latif et al 2019 | N |  |  | Y |  |  | N |  |  | N |  |  | N |  |  | N |  |  | N |  |  | N |  |  | 1 | High |
|  | Acharya et al, 2022 | Y |  |  | Y |  |  | U |  |  | U |  |  | U |  |  | U |  |  | U |  |  | Y |  |  | 3 | High |
|  | Murtaza et al, 2022 | Y |  |  | Y |  |  | Y |  |  | Y |  |  | U |  |  | U |  |  | Y |  |  | Y |  |  | 6 | Medium |

**Items and coding**

Item1–Were the criteria for inclusion in the sample clearly defined?

Item2–Were the study subjects and the setting described in detail?

Item3– Was the exposure measured in a valid and reliable way?

Item4– Were objective, standard criteria used for measurement of the condition?

Item5– Were confounding factors identified?

Item6– Were strategies to deal with confounding factors stated?

Item7– Were the outcomes measured in a valid and reliable way?

Item8– Was there appropriate statistical analysis?

Coding: Y=Yes, N=No, U=Unclear

***Note:*** *Low risk of bias (7-8); medium risk of bias (5-6), high risk of bias (1-4)*

**Table S8:** Joanna Briggs Institute risk of bias assessment for **cohort studies**

| S.No | Authors/year | Item 1 | Item 2 | Item 3 | Item 4 | Item 5 | Item 6 | Item 7 | Item8 | Item9 | Item 10 | Item 11 | Total | Judgement |
| --- | --- | --- | --- | --- | --- | --- | --- | --- | --- | --- | --- | --- | --- | --- |
|  | Arshad et al 2021 | N | U | N | Y | N | N | U | N | U | N | Y | 2 | High |
|  | Ball et al 2014 | U | Y | Y | Y | Y | Y | Y | U | Y | U | Y | 8 | Medium |
|  | Huo et al 2013 | Y | Y | Y | Y | Y | Y | Y | U | U | U | Y | 8 | Medium |
|  | Negi et al 2006 | Y | N | N | Y | N | Y | Y | U | U | Y | Y | 6 | Medium |
|  | Tanigawa et al 2021 | U | U | Y | Y | Y | Y | Y | Y | Y | Y | Y | 9 | Low |
|  | Yamashita et al 2015 | U | Y | Y | Y | Y | Y | Y | U | U | U | Y | 7 | Medium |
|  | Zhang et al 2018 | U | U | Y | Y | Y | Y | Y | U | U | U | Y | 6 | Medium |
|  | Hosain et al, 2006 | U | U | N | N | N | Y | N | Y | Y | U | Y | 4 | High |
|  | Nakamura et al, 2022 | Y | Y | Y | Y | Y | Y | Y | U | Y | U | Y | 9 | Low |
|  | Tanigawa et al, 2023 | Y | Y | Y | Y | Y | Y | Y | Y | U | Y | Y | 10 | Low |
|  | Bera et al, 2023 | Y | Y | U | N | N | Y | U | U | U | U | N | 3 | High |
|  | Regan et al 2019 | Y | Y | Y | Y | Y | Y | Y | Y | U | U | Y | 9 | Low |

**Items and coding**

Item1– Were the two groups similar and recruited from the same population?

Item2– Were the exposures measured similarly to assign people to both exposed and unexposed groups?

Item3– Was the exposure measured in a valid and reliable way?

Item4– Were confounding factors identified?

Item5– Were strategies to deal with confounding factors stated?

Item6– Were the groups/participants free of the outcome at the start of the study (or now of exposure)?

Item7– Were the outcomes measured in a valid and reliable way?

Item8– Was the follow up time reported and sufficient to be long enough for outcomes to occur?

Item9– Was follow up complete, and if not, were the reasons to loss to follow up described and explored?

Item10–Were strategies to address incomplete follow up utilized?

Item11–Was appropriate statistical analysis used?

Coding: Y=Yes, N=No, U=Unclear

***Note:*** *Low risk of bias (9-11); medium risk of bias (6-8), high risk of bias (1-5)*

**Table S9:** Joanna Briggs Institute risk of bias assessment for **case-control studies**

| S.No | Authors/year | Item 1 | Item 2 | Item 3 | Item 4 | Item 5 | Item 6 | Item 7 | Item8 | Item9 | Item 10 | Total | Judgement |
| --- | --- | --- | --- | --- | --- | --- | --- | --- | --- | --- | --- | --- | --- |
|  | Hanif et al 2019 | U | N | U | U | N | Y | Y | N | U | Y | 3 | High |
|  | Memon et al 2005 | Y | N | Y | Y | U | Y | Y | Y | Y | Y | 8 | Low |
|  | Nagargoje et al 2011 | U | Y | Y | Y | Y | Y | Y | Y | Y | Y | 9 | Low |
|  | Peters et al 2001 | Y | Y | Y | Y | Y | Y | Y | Y | U | Y | 9 | Low |
|  | Zhang et al 2012 | U | Y | Y | Y | U | Y | Y | Y | Y | Y | 8 | Low |
|  | Reddy et al 2022 | Y | U | U | U | Y | N | N | N | U | U | 2 | High |

**Items and coding**

Item1– Were the groups comparable other than the presence of disease in cases or the absence of disease in controls?

Item2– Were cases and controls matched appropriately?

Item3– Were the same criteria used for identification of cases and controls?

Item4– Was exposure measured in a standard, valid and reliable way?

Item5– Was exposure measured in the same way for cases and controls?

Item6– Were confounding factors identified?

Item7– Were strategies to deal with confounding factors stated?

Item8– Were outcomes assessed in a standard, valid and reliable way for cases and controls?

Item9– Was the exposure period of interest long enough to be meaningful?

Item10– Was appropriate statistical analysis used?

Coding: Y=Yes, N=No, U=Unclear

***Note:*** *Low risk of bias (8-10); medium risk of bias (5-7), high risk of bias (1-4*

**Table s10**: Stratified analysis of pooled effects of short interpregnancy interval and short birth interval on adverse perinatal outcomes

| Classification | Number of papers | Reported perinatal outcomes | | | | | | | | | | | |
| --- | --- | --- | --- | --- | --- | --- | --- | --- | --- | --- | --- | --- | --- |
|  |  | **LBW** | | | **Preterm birth** | | | **SGA** | | | **Neonatal mortality** | | |
|  |  | Pooled OR (95%CI) | Heterogeneity, *p* | Meta-regression, *p* | Pooled OR (95%CI) | Heterogeneity, *p* | Meta-regression, *p* | Pooled OR (95%CI) | Heterogeneity, *p* | Meta-regression, *p* | Pooled OR (95%CI) | Heterogeneity, *p* | Meta-regression, *p* |
| **Short IPI** |  |  |  |  |  |  |  |  |  |  |  |  |  |
| <6 months | 7 | 1.43 (1.22-1.68) | 92.6 | 0.25 | 1.50 (1.38-1.63) | 72.7 | 0.24 | 1.24 (1.09-1.41) | 90.7 | 0.13 |  |  |  |
| <12 months | 1 | 1.23 (1.41-1.80) | NA | NA | 1.68 (1.33-2.13) | 12.2 |  | NR |  |  | NR |  |  |
| <18 months | 4 | 3.22 (0.95-10.95) | 95.1 | 0.32 | 3.22 (0.95-10.95) | 95.1 | 0.14 | NR |  |  |  |  |  |
| < 24 months | 1 | 1.98 (1.46-2.69) | NA | NA |  |  |  | NR |  |  | NR |  |  |
| <23 months | 1 | 4.85 (1.92-12.25) | 0.0 | NA | NR |  |  | NR |  |  | NR |  |  |
| **Short BI** |  |  |  |  |  |  |  |  |  |  |  |  |  |
| <6 months | 1 | NR |  |  | NR |  |  | NR |  |  | 0.97 (0.66-1.45) | 0.0 | 0.0 |
| <12 months | 2 | 4.84 (4.42-5.30) | 0.0 | 0.0 | NR |  |  | NR |  |  | NR |  |  |
| < 18 months | 2 | NR |  |  | NR |  |  | NR |  |  | 2.35 (1.51-3.64) | 91.3 | 0.08 |
| <24 months | 10 | 1.18 (0.67-20.7) | 81.4 | 0.05 | NR |  |  | NR |  |  | 1.64 (1.47-1.82) | 0.0 | 0.0 |
| < 33 months | 1 | 1.01 (0.98-1.05) | 0.0 | 0.0 | NR |  |  | NR |  |  | 2.10 (1.24-3.55) | 0.0 | 0.0 |

*NA: not applicable; NR: not reported*

**Supplementary Figures**


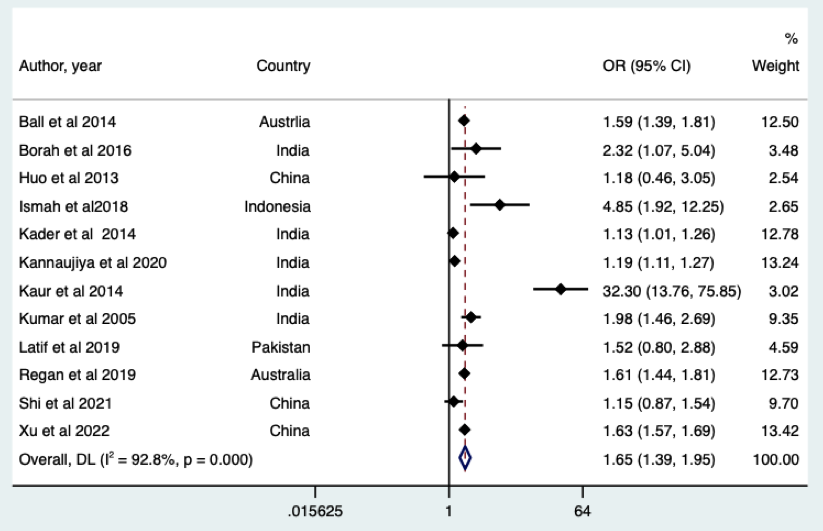


Supplementary Figure 1: Effects of short inter-pregnancy interval on low birth weight

**Figure 1A: Trim and fill estimate without (a) and with (b) missing studies**


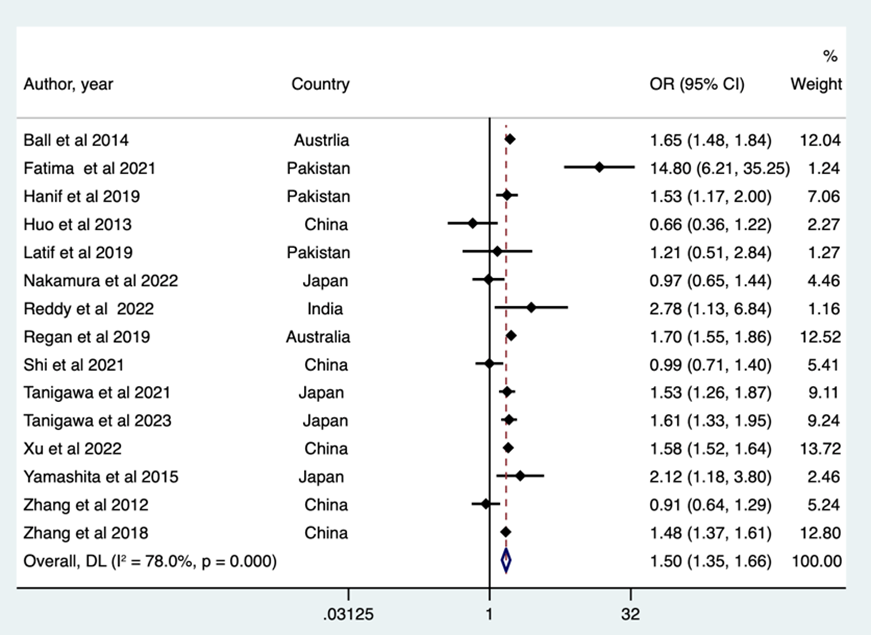


**Figure 2: Effects of short inter-pregnancy interval on preterm birth**

Figure 2A: **Trim and fill estimate without (a) and with (b) missing studies**


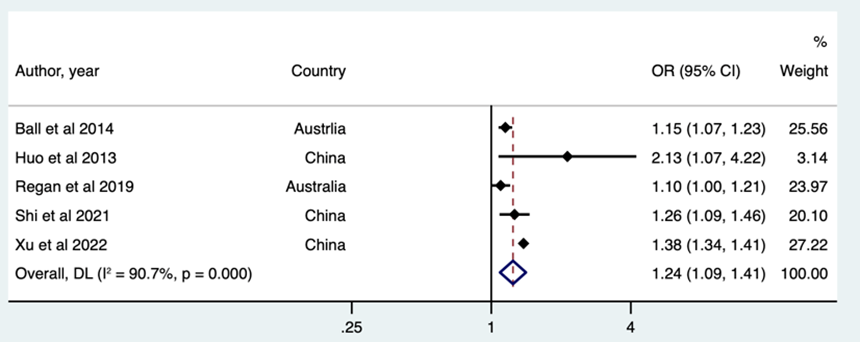


Figure 3: **Effects of short inter-pregnancy interval on small for gestational age**


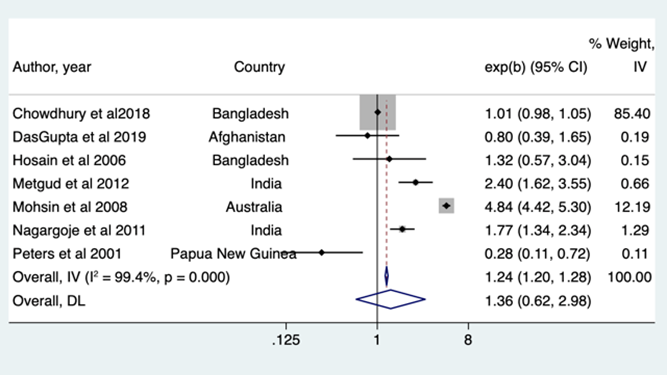


Figure 4: **Effects of short birth interval on low birth weight**

Figure 4A: **Trim and fill estimate without (a) and with (b) missing studies**


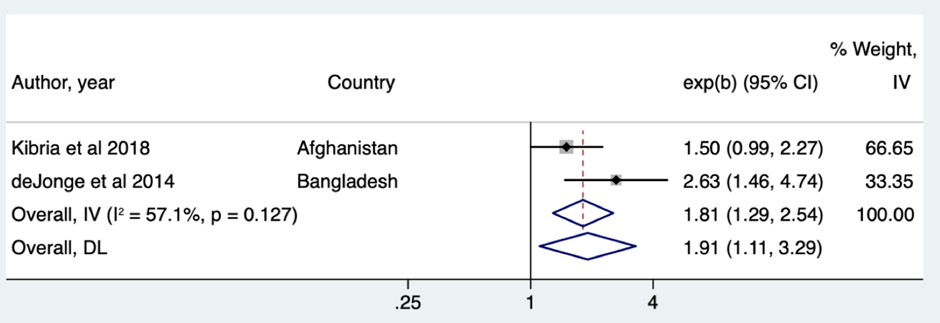


Figure 5**: Effects of short birth interval on early neonatal mortality**


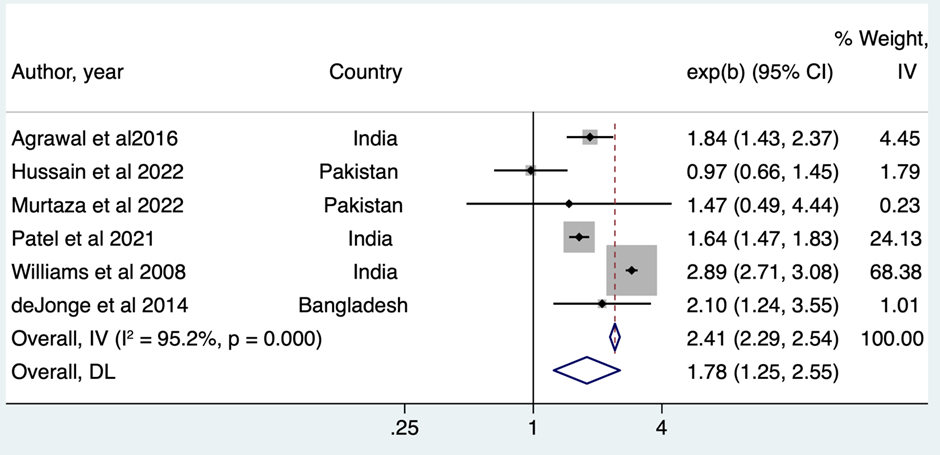


Figure 6: **Effects of short birth interval on neonatal mortality**

**Figure 6A: Trim and fill estimate without (a) and with (b) missing studies**


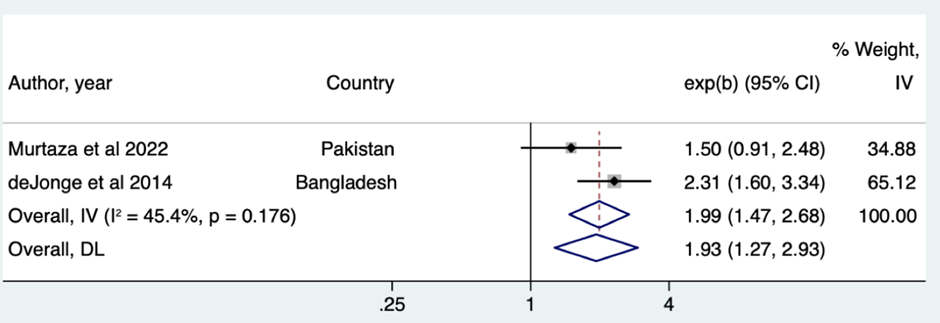


Figure 7: **Effects of short birth interval on perinatal mortality**


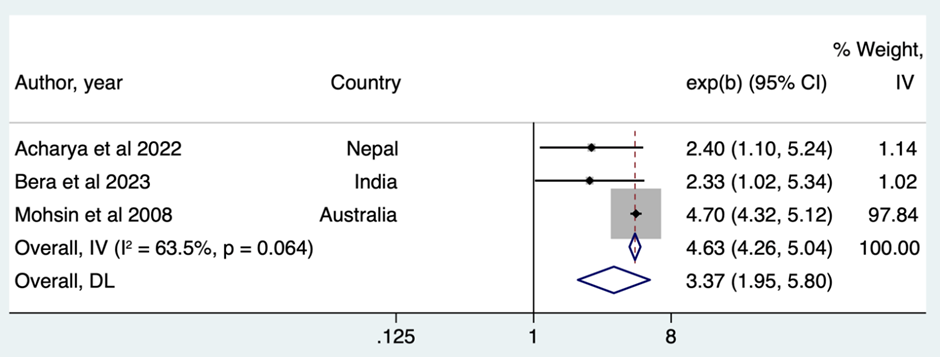


Figure 8: Effects of short birth interval on preterm birth


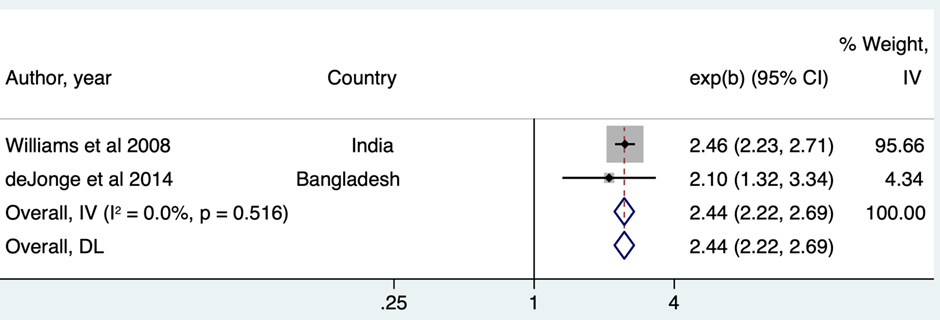


Figure 14: Effects of short birth interval on stillbirths


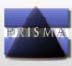
**PRISMA 2020 Checklist**

| **Section and Topic** | **Item #** | **Checklist item** | **Location where item is reported** |
| --- | --- | --- | --- |
| **TITLE** | | |  |
| Title | 1 | Identify the report as a systematic review. | Page 1 |
| **ABSTRACT** | | |  |
| Abstract | 2 | See the PRISMA 2020 for Abstracts checklist. | Page 1 |
| **INTRODUCTION** | | |  |
| Rationale | 3 | Describe the rationale for the review in the context of existing knowledge. | Page 2-3 |
| Objectives | 4 | Provide an explicit statement of the objective(s) or question(s) the review addresses. | Page 4 |
| **METHODS** | | |  |
| Eligibility criteria | 5 | Specify the inclusion and exclusion criteria for the review and how studies were grouped for the syntheses. | Page 4 |
| Information sources | 6 | Specify all databases, registers, websites, organisations, reference lists and other sources searched or consulted to identify studies. Specify the date when each source was last searched or consulted. | Page 4 |
| Search strategy | 7 | Present the full search strategies for all databases, registers and websites, including any filters and limits used. | Page 5 and Supplementary Table S1-5 |
| Selection process | 8 | Specify the methods used to decide whether a study met the inclusion criteria of the review, including how many reviewers screened each record and each report retrieved, whether they worked independently, and if applicable, details of automation tools used in the process. | Page 5 |
| Data collection process | 9 | Specify the methods used to collect data from reports, including how many reviewers collected data from each report, whether they worked independently, any processes for obtaining or confirming data from study investigators, and if applicable, details of automation tools used in the process. | Page 5 |
| Data items | 10a | List and define all outcomes for which data were sought. Specify whether all results that were compatible with each outcome domain in each study were sought (e.g. for all measures, time points, analyses), and if not, the methods used to decide which results to collect. | Page 5 |
|  | 10b | List and define all other variables for which data were sought (e.g. participant and intervention characteristics, funding sources). Describe any assumptions made about any missing or unclear information. | Page 6 |
| Study risk of bias assessment | 11 | Specify the methods used to assess risk of bias in the included studies, including details of the tool(s) used, how many reviewers assessed each study and whether they worked independently, and if applicable, details of automation tools used in the process. | Page 6 |
| Effect measures | 12 | Specify for each outcome the effect measure(s) (e.g. risk ratio, mean difference) used in the synthesis or presentation of results. | Page 6 |
| Synthesis methods | 13a | Describe the processes used to decide which studies were eligible for each synthesis (e.g. tabulating the study intervention characteristics and comparing against the planned groups for each synthesis (item #5)). | Page 6 |
|  | 13b | Describe any methods required to prepare the data for presentation or synthesis, such as handling of missing summary statistics, or data conversions. | Page 6 |
|  | 13c | Describe any methods used to tabulate or visually display results of individual studies and syntheses. | Page 6 |
|  | 13d | Describe any methods used to synthesize results and provide a rationale for the choice(s). If meta-analysis was performed, describe the model(s), method(s) to identify the presence and extent of statistical heterogeneity, and software package(s) used. | Page 6 |
|  | 13e | Describe any methods used to explore possible causes of heterogeneity among study results (e.g. subgroup analysis, meta-regression). | Page6 |
|  | 13f | Describe any sensitivity analyses conducted to assess robustness of the synthesized results. | Page6 |
| Reporting bias assessment | 14 | Describe any methods used to assess risk of bias due to missing results in a synthesis (arising from reporting biases). | Page6 |
| Certainty assessment | 15 | Describe any methods used to assess certainty (or confidence) in the body of evidence for an outcome. | NA |
| **RESULTS** | | |  |
| Study selection | 16a | Describe the results of the search and selection process, from the number of records identified in the search to the number of studies included in the review, ideally using a flow diagram. | Page 7 |
|  | 16b | Cite studies that might appear to meet the inclusion criteria, but which were excluded, and explain why they were excluded. | Page 7 |
| Study characteristics | 17 | Cite each included study and present its characteristics. | Page 8 and supplementary TableS6 |
| Risk of bias in studies | 18 | Present assessments of risk of bias for each included study. | Supplementary TableS7-9 |
| Results of individual studies | 19 | For all outcomes, present, for each study: (a) summary statistics for each group (where appropriate) and (b) an effect estimate and its precision (e.g. confidence/credible interval), ideally using structured tables or plots. | Page 8-9 and supplementary Table S6 |
| Results of syntheses | 20a | For each synthesis, briefly summarise the characteristics and risk of bias among contributing studies. | Page 8 |
|  | 20b | Present results of all statistical syntheses conducted. If meta-analysis was done, present for each the summary estimate and its precision (e.g. confidence/credible interval) and measures of statistical heterogeneity. If comparing groups, describe the direction of the effect. | Page 8-9 |
|  | 20c | Present results of all investigations of possible causes of heterogeneity among study results. | Supplementary Table S10 |
|  | 20d | Present results of all sensitivity analyses conducted to assess the robustness of the synthesized results. | Page10-11 (Table 1 and 2) |
| Reporting biases | 21 | Present assessments of risk of bias due to missing results (arising from reporting biases) for each synthesis assessed. | Page10-11 (Table 1 and 2) |
| Certainty of evidence | 22 | Present assessments of certainty (or confidence) in the body of evidence for each outcome assessed. | NA |
| **DISCUSSION** | | |  |
| Discussion | 23a | Provide a general interpretation of the results in the context of other evidence. | Page 10 |
|  | 23b | Discuss any limitations of the evidence included in the review. | Page 10 |
|  | 23c | Discuss any limitations of the review processes used. | Page 12 |
|  | 23d | Discuss implications of the results for practice, policy, and future research. | Page 12-13 |
| **OTHER INFORMATION** | | |  |
| Registration and protocol | 24a | Provide registration information for the review, including register name and registration number, or state that the review was not registered. | PROSPERO(CRD42023432913 |
|  | 24b | Indicate where the review protocol can be accessed, or state that a protocol was not prepared. | Under review with BMJ open |
|  | 24c | Describe and explain any amendments to information provided at registration or in the protocol. | NA |
| Support | 25 | Describe sources of financial or non-financial support for the review, and the role of the funders or sponsors in the review. | None |
| Competing interests | 26 | Declare any competing interests of review authors. | None |
| Availability of data, code and other materials | 27 | Report which of the following are publicly available and where they can be found: template data collection forms; data extracted from included studies; data used for all analyses; analytic code; any other materials used in the review. | All included in the manuscript |

**Reference**

1 Arshad A, Javaid MK, Rehman A. Comparison of Perinatal Outcome (Low Birth Weight, Preterm Delivery) in Women with < 6 Month Versus 12-17 Months of Interpregnancy Birth Interval. PAKISTAN JOURNAL OF MEDICAL & HEALTH SCIENCES. 2021;15:2742-5.

2 Ball SJ, Pereira G, Jacoby P, de Klerk N, Stanley FJ. Re-evaluation of link between interpregnancy interval and adverse birth outcomes: retrospective cohort study matching two intervals per mother. Bmj. 2014;349:g4333.

3 Borah M, Agarwalla R. Maternal and socio-demographic determinants of low birth weight (LBW): A community-based study in a rural block of Assam. Journal of Postgraduate Medicine. 2016;62:178-81.

4 Chowdhury M, Dibley MJ, Alam A, Huda TM, Raynes-Greenow C. Household Food Security and Birth Size of Infants: Analysis of the Bangladesh Demographic and Health Survey 2011. Curr. 2018;2:nzy003.

5 Das Gupta R, Swasey K, Burrowes V, Hashan MR, Al Kibria GM. Factors associated with low birth weight in Afghanistan: a cross-sectional analysis of the demographic and health survey 2015. BMJ Open. 2019;9:e025715.

6 de Jonge HCC, Azad K, Seward N, Kuddus A, Shaha S, Beard J, et al. Determinants and consequences of short birth interval in rural Bangladesh: a cross-sectional study. BMC Pregnancy Childbirth. 2014;14:427.

7 Fatima M, Naz U, Hira AK, Habib A, Kazi PS, Majeed H. Association Between Pre-Term Labour and Inter Pregnancy Interval. PAKISTAN JOURNAL OF MEDICAL & HEALTH SCIENCES. 2021;15:3137-9.

8 Hanif A, Ashraf T, Pervaiz MK, Güler N. Maternal, fetal and neonatal risk factors for preterm birth in parity > 1. Pakistan Paediatric Journal. 2019;43:270-7.

9 Hosain GM, Chatterjee N, Begum A, Saha SC. Factors associated with low birthweight in rural Bangladesh. J Trop Pediatr. 2006;52:87-91.

10 Huo X-X, Gao E-S, Cheng Y-M, Luo L, Liang H, Huang G-Y, et al. Effect of interpregnancy interval after a mifepristone-induced abortion on neonatal outcomes in subsequent pregnancy. Contraception. 2013;87:38-44.

11 Hussain R. Risk factors for neonatal mortality in low-income population subgroups in Karachi, Pakistan. Community Genet. 2002;5:249-56.

12 Ismah Z, Tjekyan S, Novrikasari. Incident of Low Fetal Weight in Relation to the Interval Between Pregnancies. 2018. p. 6677-80.

13 Kader M, Perera NKPP. Socio-economic and nutritional determinants of low birth weight in India. N A J Med Sci (Hamilt). 2014;6:302-8.

14 Kannaujiya AK, Kumar K, Upadhyay AK, McDougal L, Raj A, Singh A. Short interpregnancy interval and low birth weight births in India: Evidence from National Family Health Survey 2015-16. SSM Popul Health. 2020;12:100700.

15 Kaur S, Upadhyay AK, Srivastava DK, Srivastava R, Pandey ON. Maternal correlates of birth weight of newborn: A hospital based study. Indian Journal of Community Health. 2014;26:187-91.

16 Kibria GMA, Burrowes V, Choudhury A, Sharmeen A, Ghosh S, Mahmud A, et al. Determinants of early neonatal mortality in Afghanistan: an analysis of the Demographic and Health Survey 2015. Global health. 2018;14:47.

17 Kumar S, Dabral M, Jaiswal K, Singh CM. A STUDY ON MATERNAL FACTORS AND PREGNANCY OUTCOME IN MEDICAL COLLEGE HOSPITAL OF JHANSI CITY. INDIAN JOURNAL OF COMMUNITY HEALTH. 2005;17:5-9.

18 Memon Y, Sheikh S, Memon A. MATERNAL RISK FACTORS AFFECTING BIRTH WEIGHT OF NEWBORN. JOURNAL OF THE LIAQUAT UNIVERSITY OF MEDICAL AND HEALTH SCIENCES. 2005;4:94-9.

19 Metgud CS, Naik VA, Mallapur MD. Factors affecting birth weight of a newborn--a community based study in rural Karnataka, India. PLoS ONE. 2012;7:e40040.

20 Mohsin M, Jalaludin B. Influence of previous pregnancy outcomes and continued smoking on subsequent pregnancy outcomes: an exploratory study in Australia. Bjog. 2008;115:1428-35.

21 Nagargoje MM, Chaudhary SS, Deshmukh JS, Gupta SC, Misra SK. A case control study for risk factors of low birth weight in Nagpur city of Maharashtra. INDIAN JOURNAL OF COMMUNITY HEALTH. 2011;23:4-7.

22 Negi KS, Kandpal SD, Kukreti M. Epidemiological factors affecting low birth weight. JK Science. 2006;8:31-4.

23 Patel KK, Kumar M. Differential and Determinants of Neonatal Mortality: A Comparative Study in Northern and Southern Regions of India. Indian J. 2021;46:405-10.

24 Peters HR, Vince JD, Friesen H. Low birthweight at a Papua New Guinea highlands hospital. J Trop Pediatr. 2001;47:17-23.

25 Regan AK, Ball SJ, Warren JL, Malacova E, Padula A, Marston C, et al. A Population-Based Matched-Sibling Analysis Estimating the Associations Between First Interpregnancy Interval and Birth Outcomes. Am J Epidemiol. 2019;188:9-16.

26 Shi G, Zhang B, Kang Y, Dang S, Yan H. Association of Short and Long Interpregnancy Intervals with Adverse Birth Outcomes: Evidence from a Cross-Sectional Study in Northwest China. Int J Gen Med. 2021;14:2871-81.

27 Tanigawa K, Ikehara S, Cui M, Kawanishi Y, Kimura T, Ueda K, et al. Association between interpregnancy interval and risk of preterm birth and its modification by folate intake: the Japan Environment and Children's Study. J Epidemiol. 2021;22:22.

28 Williams EK, Hossain MB, Sharma RK, Kumar V, Pandey CM, Baqui AH. Birth interval and risk of stillbirth or neonatal death: findings from rural north India. J Trop Pediatr. 2008;54:321-7.

29 Xu T, Miao H, Chen Y, Luo L, Guo P, Zhu Y. Association of Interpregnancy Interval With Adverse Birth Outcomes. JAMA netw. 2022;5:e2216658.

30 Yamashita M, Hayashi S, Endo M, Okuno K, Fukui O, Mimura K, et al. Incidence and risk factors for recurrent spontaneous preterm birth: A retrospective cohort study in Japan. Journal of Obstetrics & Gynaecology Research. 2015;41:1708-14.

31 Zhang L, Shen S, He J, Chan F, Lu J, Li W, et al. Effect of Interpregnancy Interval on Adverse Perinatal Outcomes in Southern China: A Retrospective Cohort Study, 2000-2015. Paediatr Perinat Epidemiol. 2018;32:131-40.

32 Zhang Y-P, Liu X-H, Gao S-H, Wang J-M, Gu Y-S, Zhang J-Y, et al. Risk factors for preterm birth in five Maternal and Child Health hospitals in Beijing. PLoS ONE. 2012;7:e52780.

33 Agrawal S, Agrawal PK, Williams EK, Darmstadt GL, Kumar V, Kiran U, et al. Rural community-based maternal and newborn interventions on prevention of neonatal morality. Nova Science Publishers, Inc.; 2016. p. 17-34.

34 Asif MF, Meherali S, Abid G, Khan MS, Lassi ZS. Predictors of Child's Health in Pakistan and the Moderating Role of Birth Spacing. Int J Environ Res Public Health. 2022;19:03.

35 Latif L, Iqbal UJ. Fetomaternal Outcomes of Short Inter-pregnancy Interval. PAKISTAN JOURNAL OF MEDICAL & HEALTH SCIENCES. 2019;13:424-6.

36 Nakamura Y, Tsuda H, Masahashi Y, Nakamura T, Suzuki M, Fukuhara N, et al. Impact of the interpregnancy interval after cesarean delivery on subsequent perinatal risks: a retrospective study. Arch Gynecol Obstet. 2022:7.

37 Acharya D, Gautam S, Poder TG, Lewin A, Gaussen A, Lee K, et al. Maternal and dietary behavior-related factors associated with preterm birth in Southeastern Terai, Nepal: A cross sectional study. Front. 2022;10:946657.

38 Tanigawa K, Ikehara S, Cui M, Kawanishi Y, Kimura T, Ueda K, et al. Association Between Interpregnancy Interval and Risk of Preterm Birth and Its Modification by Folate Intake: The Japan Environment and Children's Study. J Epidemiol. 2023;33:113-9.

39 Reddy KM, Ravula SR, Palakollu S, Betha K. Prevalence of preterm birth and perinatal outcome: A rural tertiary teaching hospital-based study. J Fam Med Prim Care. 2022;11:3909-14.

40 Murtaza K, Saleem Z, Jabeen S, Alzahrani AK, Kizilbash N, Soofi SB, et al. Impact of interpregnancy intervals on perinatal and neonatal outcomes in a multiethnic Pakistani population. J Trop Pediatr. 2022;68:06.

41 Bera M, Chaudhury N, Samanta S. Interpregnancy Interval Effect on Perinatal Outcome- A Prospective Observational Study. J Clin Diagn Res. 2023;17:QC1-QC4.
